# STOML2 interacts with PHB through activating MAPK signaling pathway to promote colorectal Cancer proliferation

**DOI:** 10.1186/s13046-021-02116-0

**Published:** 2021-11-15

**Authors:** Wenhui Ma, Yuehong Chen, Wenjun Xiong, Wenyi Li, Zhuoluo Xu, Ying Wang, Zhigang Wei, Tingyu Mou, Zhaokun Wu, Mingzhen Cheng, Yini Zou, Yu Zhu, Weijie Zhou, Feng Liu, Yan Geng

**Affiliations:** 1 Department of Gastrointestinal Surgery, Shunde Hospital, Southern Medical University (The First People’s Hospital of Shunde), No. 1 Jiazi Road, Lunjiao, Shunde District, Foshan City, 528308 Guangdong Province China; 2 Departments of General Surgery, Nanfang Hospital, Southern Medical University, Guangzhou, Guangdong China; 3 Guangdong Provincial Key Laboratory of Precision Medicine for Gastrointestinal Tumor, Guangzhou, China; 4 Department of Pathology, School of Basic Medical Sciences, Southern Medical University, Guangzhou, 510515 Guangdong China; 5 Guangdong Provincial Key Laboratory of Molecular Tumor Pathology, Guangzhou, Guangdong China; 6 Departments of Oncology, Nanfang Hospital, Southern Medical University, Guangzhou, Guangdong China; 7 Department of Gastrointestinal Surgery, Guangdong Provincial Hospital of Chinese Medicine, the Second Affiliated Hospital of Guangzhou University of Chinese, Guangzhou, Guangdong China; 8 Department of Colorectal and Anal Surgery, Guangzhou First People’s Hospital, School of Medicine, South China University of Technology, Guangzhou, 510180 Guangdong China

**Keywords:** Colorectal cancer, STOML2, PHB, MAPK signaling pathway, Proliferation

## Abstract

**Background:**

Highly expressed STOML2 has been reported in a variety of cancers, yet few have detailed its function and regulatory mechanism. This research aims to reveal regulatory mechanism of STOML2 and to provide evidence for clinical therapeutics, via exploration of its role in colorectal cancer, and identification of its interacting protein.

**Methods:**

Expression level of STOML2 in normal colon and CRC tissue from biobank in Nanfang Hospital was detected by pathologic methods. The malignant proliferation of CRC induced by STOML2 was validated via gain-of-function and loss-of-function experiments, with novel techniques applied, such as organoid culture, orthotopic model and endoscopy monitoring. Yeast two-hybrid assay screened interacting proteins of STOML2, followed by bioinformatics analysis to predict biological function and signaling pathway of candidate proteins. Target protein with most functional similarity to STOML2 was validated with co-immunoprecipitation, and immunofluorescence were conducted to co-localize STOML2 and PHB. Pathway regulated by STOML2 was detected with immunoblotting, and subsequent experimental therapy was conducted with RAF inhibitor Sorafenib.

**Results:**

STOML2 was significantly overexpressed in colorectal cancer and its elevation was associated with unfavorable prognosis. Knockdown of STOML2 suppressed proliferation of colorectal cancer, thus attenuated subcutaneous and orthotopic tumor growth, while overexpressed STOML2 promoted proliferation in cell lines and organoids. A list of 13 interacting proteins was screened out by yeast two-hybrid assay. DTYMK and PHB were identified to be most similar to STOML2 according to bioinformatics in terms of biological process and signaling pathways; however, co-immunoprecipitation confirmed interaction between STOML2 and PHB, rather than DTYMK, despite its highest rank in previous analysis. Co-localization between STOML2 and PHB was confirmed in cell lines and tissue level. Furthermore, knockdown of STOML2 downregulated phosphorylation of RAF1, MEK1/2, and ERK1/2 on the MAPK signaling pathway, indicating common pathway activated by STOML2 and PHB in colorectal cancer proliferation.

**Conclusions:**

This study demonstrated that in colorectal cancer, STOML2 expression is elevated and interacts with PHB through activating MAPK signaling pathway, to promote proliferation both in vitro and in vivo. In addition, combination of screening assay and bioinformatics marks great significance in methodology to explore regulatory mechanism of protein of interest.

**Supplementary Information:**

The online version contains supplementary material available at 10.1186/s13046-021-02116-0.

## Background

Colorectal cancer (CRC) is the third most prevalent malignant disease (10.2% of 18.1 million for incidence), and the second leading cause of cancer related death worldwide (9.2% of 9.6 million for mortality) [1, 2]. The 5-year overall survival rate of patients with CRC is about 56.9–57.6% [3]. Malignant proliferation, invasion-metastasis and chemo-resistance are the main causes of relapse and poor prognosis in CRC. Despite advancing diagnostic techniques and comprehensive therapy, our understanding of underlying mechanism of its malignant phenotype is still limited [4, 5]. Therefore, it is the primary task for researchers to find out accurate and effective biomarkers, explore how they function in the oncologic process of CRC, and assess their prognostic and therapeutic value.

Stomatin-like 2 (aka STOML2, SLP2), which is encoded by gene located on chromosome 9p13, had been initially cloned and identified as a novel member of stomatin family in human erythrocytes, lacking NH2-terminal hydrophobic domain [6]. Under physiological conditions, STOML2 is identified within inner mitochondria membrane and faces the intermembrane space, interacts with certain components of the inner mitochondria membrane, and serves as a regulator of biogenesis and the activity of mitochondria [7, 8].

Since Zhang et al. discovered the elevation of STOML2 in human esophageal squamous cell carcinoma in 2006 [9], researchers in the recent decade have successively discovered STOML2 as an overexpressed biomarker implicated in poor prognosis of pan-cancers, such as endometrial adenocarcinoma [10], glioma cells [11], papillary thyroid cancer [12], cervical cancer [13], epithelial ovarian cancer [14, 15], colorectal cancer [16, 17], liver cancer [18], head and neck squamous cell carcinoma [19], etc. Additionally, our research team had previously discovered the elevated expression and association with poor prognosis of STOML2 in gastric cancer [20], and furthermore revealed a positive feedback loop of STOML2 to promote gastric cancer progression [21]. Although discovery of STOML2 overexpression in multiple cancers and a handful of tracing to regulatory mechanism were reported, systemic assessment of STMOL2 is still absent. Recent decades have witnessed the thriving technology of high-throughput sequencing, along with a growing body of databases and bioinformatic techniques, presenting more and more effective methods in the exploration of molecular function and regulatory mechanism [22–24].

Herein, the present study focuses on the oncological function and regulatory mechanism of STOML2 in CRC. On the basis of STOML2 overexpression in CRC, multiple research models (including cell lines, organoids and animals) were utilized to demonstrate pivotal role of STOML2 in promoting CRC proliferation. Additionally, through yeast two-hybrid (Y2H) assay, thirteen proteins were screened out to be candidates interacting with STOML2. Following bioinformatics analysis of STOML2 and its interaction candidates in two large CRC datasets, downstream signaling pathway of STOML2 was identified, and experimental therapy was conducted.

## Materials and methods

### Patients and samples

This study was approved by the Ethics Committee of Nanfang Hospital (Guangzhou, China). Fresh and formalin-fixed tissue samples were collected from Department of Pathology, Nanfang Hospital. All patients given informed consents. None of these patients received chemotherapy or radiotherapy before operation.

### Cell culture

Human CRC cell lines (HCT116, HT29, SW480, SW620) and murine CRC cell lines (MC38, CT26) were purchased from Cell Resource Center, Shanghai Institute of Biochemistry and Cell Biology at the Chinese Academy of Sciences (Shanghai, China) and maintained at the Laboratory of Pathology, Southern Medical University (Guangzhou, China). Human cell lines were cultured in RPMI-1640 medium, and murine cell lines were cultured in DMEM medium, containing 10% fetal bovine serum (Gibco), at 37 °C, in the atmosphere of 5% CO_2_.

### Immunohistochemistry and immunofluorescent staining

For IHC staining, tissue slides were deparaffinized and hydrated, incubated with antibody anti-STOML2 (1:1000, Proteintech, Wuhan, China) and anti-Ki-67 (1:200, BD, CA, US) overnight at 4 °C. For negative controls, the antibodies were replaced with normal non-immune serum. Tissue slides were reviewed and scored by two independent observers, based on percentage of positive cells and the degree of positive staining. Positive cells at each intensity of staining were recorded on a scale of 0–3 (0, no staining; 1, weak staining = light yellow; 2, moderate staining = yellowish brown; 3, strong staining = brown). A score of ≥2 with at least 50% of malignant cells with positive STOML2 staining was classified as tumors with high expression of STOML2, and < 50% of malignant cells with nuclear staining or < 2 intensity score was classified as tumors with low expression of STOML2. For cell IF staining, a multiplexed tyramide signal amplification method (TSA; PerkinElmer, Inc., US) was performed on 4-μm sections for detection of the co-localization between STOML2 and PHB. Prior to each immunofluorescence labeling, antigens were retrieved with a single microwave step. Each labeling cycle consists of application of a primary antibody, a secondary antibody conjugated to horse radish peroxidase (HRP), and TSA conjugated to a fluorophore. Tissue slides were incubated with antibody against STOML2 and PHB for 30 min respectively. TSA conjugated fluorescein was used for STOML2 and CY5 for PHB. Images were captured using inverted confocal microscope (Olympus, Japan) and suite software. Images were processed using Image J and Photoshop CS5 software (Adobe Systems Inc., San Jose, CA).

### Establishment of stably transfected cell lines

SW620 cells were infected with STOML2-knockdown (shSTOML2) or scramble shRNA (Scr; GENECHEM, Shanghai, China). SW480 cells were infected with STOML2-overexpressed (3 × Flag-STOML2) or control lentivirus (mock; GENECHEM, Shanghai, China). MC38 and CT26 cells were infected with STOML2-knockdown (shSTOML2) or overexpression lentivirus (GENECHEM, Shanghai, China). MC38-STOML2 cell was infected with PHB-knockdown (shSTOML2) or scramble shRNA (Scr; GENECHEM, Shanghai, China). Cells were seeded in 6-well plates at a density of 2 × 10^5 cells per well, 24 h before transfection. 2 × 10^6 TU of corresponding lentivirus and 5 μg of polybrene (Merck, Darmstadt, Germany) were mixed to 1 mL serum-free medium to transfect cells. After transfection for 48 h, 1 μg/mL of puromycin (Merck, Germany) was added to each well to screen out the stably transfected cells, and the cells were then transferred to conventional medium. Transfection efficiency was confirmed by quantitative reverse transcription polymerase chain reaction (qRT-PCR) and immunoblot assay.

### Total RNA extraction and real-time quantitative PCR

Total RNA was extracted with Trizol reagent (TaKaRa, Dalian China) following manufacturer’s protocol. cDNA synthesis was performed with PrimeScript™ RT reagent Kit (TaKaRa, Dalian China)*. q*RT-PCR was carried out using SYBR Premix Ex Taq™ II (TaKaRa, Dalian China) on ABI-7500 instrument (Applied BioSystems). Data were normalized to the mean Ct values of housekeeping gene GAPDH and calculated using –ΔΔCt method to compare variation in gene expression.

### Cell proliferation, colony formation assay and transwell assay

For Cell Counting Kit-8 (CCK8) assay, cells were seeded in 96-well plates at a density of 800 or 5000 cells per well, respectively for murine or human CRC cell lines, one day before proliferation assay. As for organoids, after 5–7 days of culture, organoids were mechanically disrupted with cold media to depolymerize the Matrigel and generate organoid fragments. Fragments were digested with TrypLE™ Express (Thermo Fisher Scientific Inc., MA, US) and 800 U ml/L DNase1 (Roche, Swiss) under minute-to-minute vortexing to make a single-cell suspension. After counting, approximately 1000 fragments were plated in 20 μl Matrigel and cultured in medium. Where indicated, SW480 cells were pre-treated with 10 μM sorafenib (Sigma-Aldrich, MO, US) or equal volume of dimethylsulfoxide (DMSO) before CCK8 assay. Cell medium of each well was discarded and replaced by CCK-8 reagent 2 h before testing. Absorbance value was detected at 450 nm wavelength by Microplate Reader (Perkin Elmer, MA, US), continuously for 4 days. For colony formation assay, cells were sufficiently distributed in 6-well plates with 3 ml complete medium. After 14-day incubation at 37 °C in an atmosphere of 5% CO2, the colonies formed by single cells were fixed in 75% ethanol and stained with Giemsa for quantification. For transwell assay, 2 × 10^5 cells were seeded with 500 μl serum-free medium into transwell chamber (Corning, NY, US), which was inserted to 24 well plate. Medium containing 30% FBS was added to the bottom chamber. After 24 h incubation, cells invaded into lower *chamber* were *fi*xed in methanol, stained in crystal violet (Sigma, MO, US) and counted under microscope. All experiments were repeated for three times.

### Cell-cycle analysis

For cell-cycle analysis, ~ 10^6 indicated cells were collected and fixed in 70% ethanol in 4 °C overnight. Cells were washed with PBS and incubated with propidium iodide and RNase A solution (9:1; #KGA512, KeyGEN BioTECH, Jiangsu, China) following manufacturer instructions. Fluorescence at 488 nm wavelength was detected and analyzed using BD LSRFortessa X-20 cell analyzer and BD FACSDiva™ Software (Becton, Dickinson and Company, NJ, US).

### Immunoblot and co-immunoprecipitation assay

Total protein of cultured cells was extracted using lysis buffer (KeyGEN, Jiangsu, China), with PMSF, protease and phosphatase inhibitor reagents added according to the manufacturer’s instruction. Equal mass of protein extract was separated in sodium dodecyl sulfatepolyacrylamide gel electrophoresis (SDS-PAGE) and then transferred to PVDF membranes (Merck Millipore, MA, US). After BSA blocking for 1 h, the protein-loading membranes were incubated with primary antibody overnight at 4 °C. These membranes were then incubated with HRP-conjugated goat anti-mouse or anti-rabbit secondary antibodies (zsbio, Beijing, China) for 1 h at room temperature. Blotting images were captured and analyzed using Image Lab Software (Bio-Rad, CA, US). GAPDH was set as endogenous reference.

For immunoprecipitation assay, total protein of cultured cells was lysed and incubated with 50 μl protein-A Sepharose beads (Santa Cruz Biotechnology, TX, US), and anti-His, anti-PHB or anti-SLP2 antibodies respectively as where indicated, at 4 °C overnight with gentle mixing and anti-IgG was set as a control. Then the samples were washed and denaturized for western blotting.

Referred antibodies were purchased from commercial sources as follows: anti-STOML2 (1:1000, Proteintech, Wuhan, China), anti-GAPDH (1:1000, Proteintech, Wuhan, China), anti-PHB (1:500, Genetex, CA, US), anti-His (1:800, Proteintech, Wuhan, China), anti-IgG (Santa Cruz Biotechnology, TX, US), anti-RAF1 (1:100, Cell Signaling Technology, MA, US), anti-p-RAF1 (1:100, Cell Signaling Technology, MA, US), anti-MEK1/2 (1:200, Cell Signaling Technology, MA, US), anti-p-MEK1/2 (1:100, Cell Signaling Technology, MA, US), anti-ERK1/2 (1:500, Cell Signaling Technology, MA, US), anti-p-ERK1/2 (1:200, Cell Signaling Technology, MA, US), anti-pan-Cytokeratin (1:1000, #C2562, Sigma-Aldrich, US).

### Isolation, culture, transfection and staining of primary murine colon tumors

Colon tumors of 16-week *Apc*^*Min/+*^ mice were isolated, collected and embedded into Matrigel (BD Biosciences) at 50 μl per well in 24-well plates, following previously published methods [25]. The culture medium was DMEM/F12, supplemented with 1 unit/ml of penicillin, 1 μg/ml of streptomycin, 2.5 ng/ml of amphotericin B, 10 mmol/L HEPES, 2 mM Glutamax, 1× N2 supplement, 1× B27 supplement, and 50 ng/ml murine EGF. Culture medium was changed every 2 days and organoids were passaged by mechanical disruption once a week. Organoid transfection was conducted as described protocol [26]. Briefly, organoids were trypsinized for 10 min at 37 °C, to obtain single cell suspension. 2 × 10^6 TU of corresponding lentivirus and 5 μg of polybrene (Merck, Germany) were mixed to 1 mL serum-free medium to transfect cells. After incubated for 48 h, 1 μg/mL of puromycin (Merck, Germany) was added to each well to screen out the stably transfected cells, which were re-embedded to Matrigel afterwards. Organoid IF staining was conducted as previously described methods [27]. Briefly, organoids were grown in Matrigel plated onto an 8-well chamber slide (Lab-Tek II, 154534). After culture medium was discarded, organoids were fixed in 4% PFA-PME (50 mM PIPES, 2.5 mM MgCl_2_, 5 mM EDTA) for 20 min, then permeabilized in 0.5% Triton for 20 min and blocked in IF Buffer (PBS, 0.2% Triton, 0.05% Tween, 1% BSA) for 1 h. Chamber slide were incubated with antibody against E-cadherin and Ki-67 for 30 min. For hematoxylin-eosin (HE)/IHC staining, organoids were embedded in agarose gel before fixed in paraformaldehyde and embedded in paraffin. Where indicated, sections were stained with hematoxylin-eosin or using pan-Cytokeratin antibody targeting epithelial cells, according to standard histology methods. Images were captured using inverted confocal microscope (Olympus, Japan) and suite software. Images were processed using Image J and Photoshop CS5 software (Adobe Systems Inc., San Jose, CA).

### Mice, tumor growth assay, and sorafenib treatment for MAPK pathway inhibition

All mice experiments were approved by Animal Research Ethics Committee of Southern Medical University and proceeded in accordance with the guidelines on the care and use of animals for scientific purposes. Stable-transfected murine CRC cells (2 × 10^6) suspended in 200 μl PBS were injected subcutaneously into the left or right hind limb of 6-week-old male wild type (WT) C57BL/6 or BALB/c mice (*n* = 7/group), accordingly. Tumor size was measured every three days with a vernier caliper, and tumor volume was calculated as 0.52 × L × W^2 (cm^3; L stands for length and W for width of the tumor). For MAPK pathway inhibition, 10 μg/mL sorafenib in 200 μL PBS was injected every three days, subcutaneously into the hind limb foot where CRC cells were implanted. The treatment was started from the second week after tumor implantation. Mice were anesthetized and sacrificed at indicated time, around 4 weeks after injection. Tumors were dissected, measured and photographed, then fixed with formalin and embedded in paraffin for further immunohistological assessment.

### Orthotopic model and murine endoscopy monitoring

Murine CRC cell lines MC38 and CT26 were suspended at density of 2 × 10^6 in 50 μL PBS for each injection. Mice were anesthetized by inhaling 1.5 to 2% isoflurane (RWD Life Science Co., Ltd., Shenzhen, China). Optical colonoscopy was performed using a Karl Storz (Tuttlingen, Germany) Image 1 HD Camera System, Image 1 HUB CCU, 175 W Xenon Light Source, and Richard Wolf 1.9 mm/9.5 Fr Integrated Telescope (part number 8626.431). Indicated cells were injected into mouse colonic lamina propria under colonoscopy, at around 1 cm from anal orifice, using a custom injection needle (Hamilton Inc., 33-gauge, small Hub RN NDL, 6 in. long, point 4, 45-degree bevel, like part number 7803–05), syringe (Hamilton Inc. part number 7656–01), and transfer needle (Hamilton Inc. part number 7770–02). Tumor growth was assessed by serial endoscopy monitoring and recording carried out every 6 days after injection, and tumor size was scored by the diameter of the colonic lumen occupied by tumor [28]. Mice were anesthetized and sacrificed at indicated time, around 30 days after injection. Total colons were dissected, measured and photographed, then fixed in formalin and embedded in paraffin for further immnuohistological assessment.

### Yeast two-hybrid screening assay

Human CRC cell line SW620 was selected to construct CRC cDNA library. Following total RNA extraction, cDNA single/double strand synthesis and purification, SW620 cDNA library was co-transformed with pGADT7-Rec (Clontech) into *Saccharomyces cerevisiae* strain Y187. Transformation efficiency and titer of CRC cDNA library was verified. The full-length of STOML2 coding sequence was cloned to inframe with the Gal4 DNA binding domain of the bait plasmid pGBKT7 (Clontech) by PCR. pGBKT7-STOML2 plasmid was transformed into *Saccharomyces cerevisiae* strain Y2H to establish bait strain, and double strand library cDNA synthesized from SW620 was co-transfected with pGADT7-Rec plasmid into Y187 to establish library host strain. Bait strain was first tested to be non-toxic to Y2H, and pGBKT7-STOML2 could not activate reporter gene by itself. Bait and library host strain were then co-cultured at 30C with 30–50 rpm swirling, and after mating for 20 h, zygotes in typical three-lobed shape were present in the mating culture. Mating mixture was spread on three 100 mm media (SD/−Trp, SD/−Leu, SD/−Leu/−Trp) at dilution of 1:10, 1:100, 1:10^3,1:10^4. After 3 to 5 days, the number of colonies were screened and mating efficiency were calculated, and remaining mixture was spread on forty-four 150 mm QDO/A media (SD/−Ade/−His/−Leu/−Trp/Aba) for 3-to-5-days culture. On QDO/A media, clones over 2 mm at diameter were selected and transferred to QDO/X/A media, to underwent higher selective pressure. Blue-stained colonies on QDO/X/A media were randomly selected to verify multiple prey genes by amplification with polymerase chain reaction. These plasmids with prey genes (pGADT7-Prey) were extracted from each blue-stained monoclone, and respectively co-transfected with pGBKT7-STOML2 and pGBKT7 into competent yeast strain pGBKT7-S + pGADT7-Prey and pGBKT7 + pGADT7-Prey. To retest the interaction and exclude false positive in yeast, these two Y2H strains were spread on DDO/X and QDO/X/A conditioned media, with pGBKT7–53 + pGADT7-T set as positive control and pGBKT7-lam + pGADT7-T as negative control.

### Statistical analysis

Data were all presented as mean ± standard error of mean (SEM) unless otherwise annotated. Statistical analysis was performed by two-tail unpaired Student’s *t* test for experiments where two means were compared. Two-way analysis of variance (ANOVA) was used to compare means of three or more experimental groups. Factorial design ANOVA was used to analyze experiments with two independent variables. Gene set enrichment analysis (GSEA) was conducted using GSEA software (ver. 4.0.3, Broad Institute, MA, US), performed with KEGG (c2) or GO-BP (c5) gene set collections of the Molecular Signature Database v7.0 [29, 30]. Specifically, data from GSE14333 and GSE17538 were divided into two groups before performing GSEA, by the median expression level of each gene (STOML2 and its potential interacting protein obtained from Y2H screening) per analysis. Statistical analyses were performed using GraphPad Prism software 5.0 (CA, US) and SPSS software (Version 22.0, IL, US).

## Results

### STOML2 is highly expressed and predicts poor prognosis in CRC patients

**Fig. 1:**
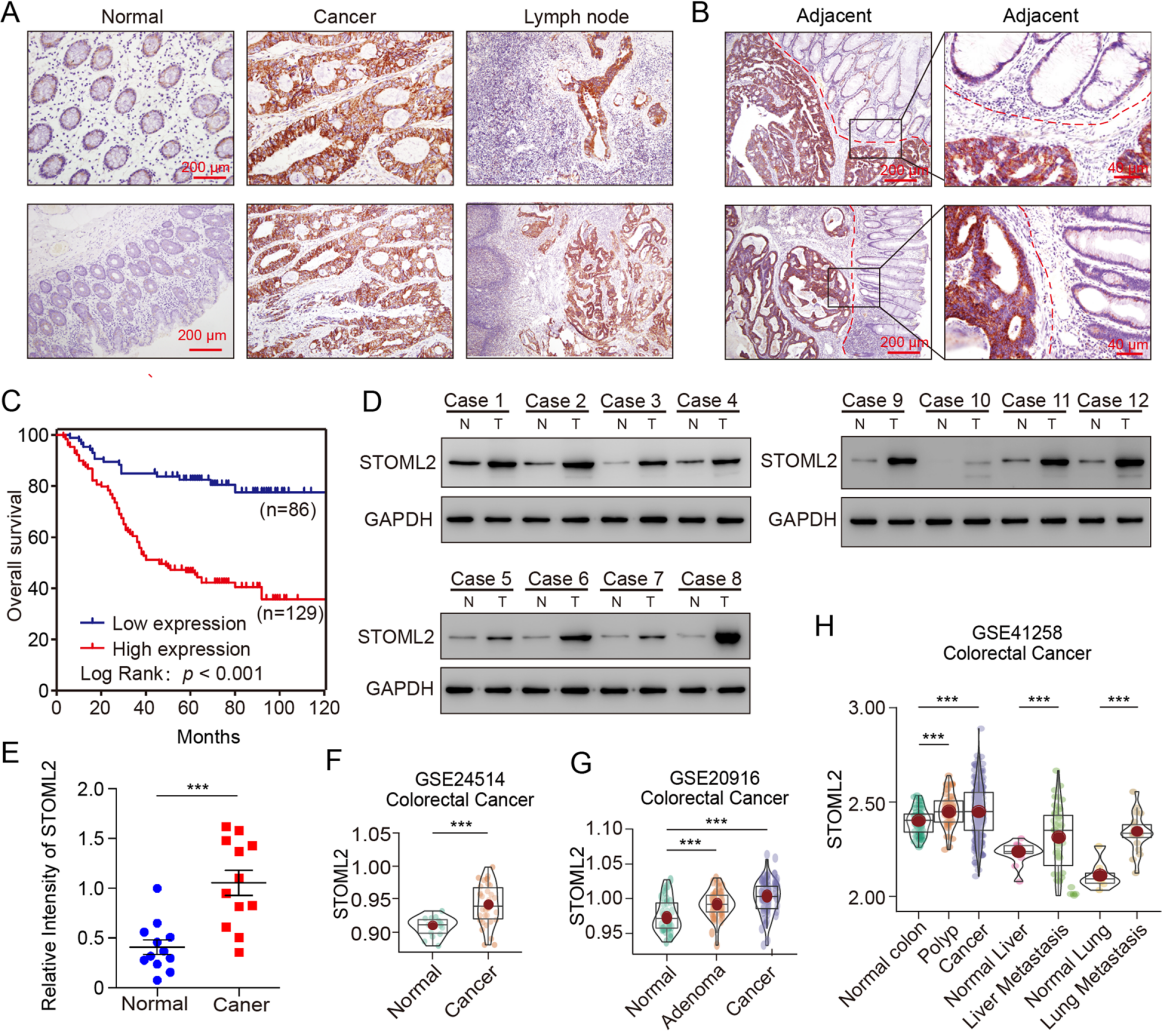
STOML2 is highly expressed and predicts poor prognosis in CRC patients. **a** Representative images of STOML2 staining in CRC, paired normal, cancer and metastatic lymph nodes tissues of internal cohort. Scale bar, 200 μm. **b** Representative images of STOML2 staining in CRC tumor-adjacent tissues of internal cohort; left: scale bar, 200 μm. right: magnified image of boxed area on the left, scale bar, 40 μm.; dotted line outlines invasive front. **c** Kaplan-Meier curves of CRC patients’ overall survival with high or low STOML2 expression level of internal cohort. ***P < 0.001, log-rank test. **d**-**e** Immunoblot of STOML2 and GAPDH in paired normal and tumor tissue of twelve CRC cases from internal cohort; (d) N, normal colon; T, tumor. (e) Relative intensity of STOML2 immunoblot assay, ***P < 0.001, *t* test. **f**-**h** External validation of STOML2 expression in different cancers from GEO database. (f) GSE24514 dataset, CRC, normal tissue vs. cancer (*n* = 15 vs. 34). (g) GSE20916 dataset, CRC, normal tissue vs. adenoma vs. cancer (*n* = 34 vs. 54 vs. 66). (h) GSE41258 dataset, CRC, normal colon polyp vs. cancer (*n* = 54 vs. 49 vs. 186), normal liver vs. liver metastasis (*n* = 13 vs. 47), normal lung vs. lung metastasis (*n* = 7 vs. 20). Data was presented as means ± SEM from five independent experiments. *** *P* < 0.001, *t* test

Following discovery of overexpression of STOML2 in gastric cancer and investigation into specific molecular mechanism of this gene afterwards [20, 21], we were intrigued whether STOML2 presented similar overexpressed profile and oncologic effect in CRC. Therefore, 215 CRC patients were randomly selected from bio-bank in General Surgery, Nanfang Hospital, as internal validation of STOML2 expression on tissue level. Immunochemistry of patients’ resected tissue showed relatively high STOML2 expression in tumor and metastatic lymph nodes, in comparison with normal tissue (Fig. 1A). In tumor-adjacent region, distinction of STOML2 expression between tumor and normal tissue was more visible, where a legible margin could be drawn (Fig. 1B). Immuno-blotting assay in twelve samples and paired tissues also confirmed higher STOML2 protein expression in tumor (Fig. 1D). Based on STOML2 expression level in tissue, these patients were then divided into high or low expression group. Statistical analyses showed significant difference in tumor clinicopathological characteristics between two groups, such as T stage, lymph node metastasis, distant metastasis, AJCC classification (Table 1). Notably, 10-year follow-up witnessed unfavorable prognosis in survival for those who were detected high STOML2 expression in resected tissue between two groups (Fig. 1C; log-rank *P* < .001). Analyses of five public datasets from Gene Expression Omnibus (GEO) in various cancer types also demonstrated common elevated expression profile of STOML2, especially in CRC and related metastatic lesions in liver and lung (Fig. 1F-H, Supplementary Fig. 1B & C). BioGPS database [31] also supported significant difference in STOML2 expression between normal colon and CRC (Supplementary Fig. 1A). These results suggest that, STOML2 is overexpressed in CRC, correlates with unfavorable clinicopathological characteristics and poor prognosis, which hints that it contributes to malignant phenotypes of CRC and is worth further study.

**Table 1:** Correlations between STOML2 expression and clinicopathological parameters of CRC patients

		STOML2 expression		***p*** value^*^
Variables	All cases (***n*** = 215)	Low (%) (***n*** = 86)	High (%) (***n*** = 129)	
**Sex**				0.569
Male	132	55 (33.8)	77 (66.2)	
Female	83	31 (34.9)	52 (65.1)	
**Age**				0.405
< 61	103	38 (35.6)	65 (64.4)	
≥61	112	48 (32.8)	64 (67.2)	
**Differentiation**				0.085
Well	81	40 (37.0)	41 (63.0)	
Moderate	105	37 (36.2)	68 (63.8)	
Poor and undifferentiated	29	9 (28.1)	20 (71.9)	
**T stage**				0.026
T1 + T2	30	18 (55.0)	12 (45.0)	
T3 + T4	185	68 (29.9)	117 (70.1)	
**Lymph node metastasis**				< 0.0001
Absent (N0)	114	62 (52.0)	52 (48.0)	
Present (N1–2)	101	24 (20.9)	77 (79.1)	
**Distant metastasis**				0.008
Absent (M0)	168	60(38.2)	108 (61.8)	
Present (M1)	23	2 (6.7)	21 (93.3)	
**AJCC classification**				< 0.0001
I	24	15	9	
II	88	47	41	
III	80	22	58	
IV	23	2	21	

^*^p values of Sex, Age, Differentiation, T stage, Lymph node metastasis, Distant metastasis, AJCC classification, were calculated by χ^2^ test

### STOML2 promotes CRC cell proliferation and colony formation in vitro

**Fig. 2:**
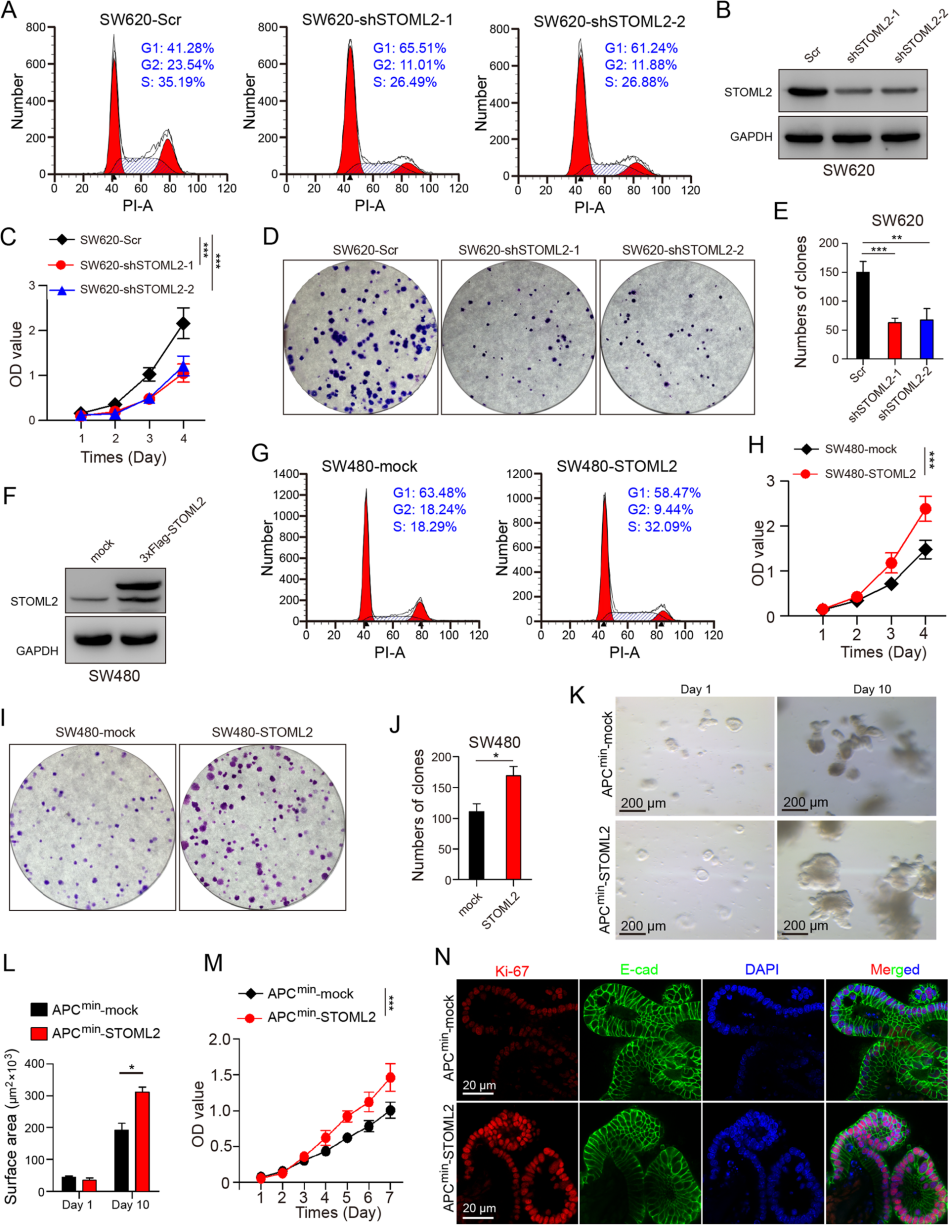
STOML2 promotes CRC cell proliferation and colony formation in vitro. **a** Cell-cycle distribution analysis of STOML2-knockdown SW620 and control. Fraction of cells in G1/G2/S phase was shown. **b** Immunoblot of STOML2 and GAPDH in SW620 transfected with STOML2-knockdown lentivirus (SW620-shSTOML2) and control group (SW620-scr). **c** CCK8 proliferation assay. Indicated cells were seeded 5000 per well in 96-well culture-plates and OD values were measured by day. Data are presented as means ± SEM from five independent experiments. ***P < 0.001, two-way ANOVA. **d** Colony formation of SW620-shSTOML2 and -scr. Indicated cells were seeded 5000 per well in 6 wells culture-plates. Giemsa staining was carried out after 14 days of culture. **e** Number of colonies formed on each plate. Data presented as means ± SEM from three independent experiments. ***P < 0.001, **P < 0.01, one-way ANOVA. **f** Immunoblot assay of indicated protein in SW480 transfected with STOML2-overexpressed lentivirus (SW480-STOML2) and control group (SW620-mock). **g** Cell-cycle distribution analysis of STOML2-overexpressed SW480 and control. Fraction of cells in G1/G2/S phase was shown. **h** CCK8 proliferation assay. Indicated cells were seeded 5000/well in 96-well culture-plates and OD values were measured by day. Data was presented as means ± SEM from five independent experiments. ***P < 0.001, two-way ANOVA. **i** Colony formation of SW480-STOML2 and -mock. 5000 indicated cells were seeded in 6 wells culture-plates. **j** Number of colonies formed on each plate. Data presented as means ± SEM from five independent experiments. *P < 0.05, *t* test. **k-n** Organoid culture, CCK8 assay and IF staining of STOML2-overexpressed organoid derived from intestinal tumor of *APC*^*Min/+*^ mice (*APC*^*min*^-STOML2) and control group (*APC*^*min*^-mock). (k) Representative images of indicated groups of organoids on first and tenth day of culture. Scale bar, 200 μm. (l) Calculated surface area (μm^2^ × 10^3^) of indicated groups of organoids. (m)CCK8 proliferation assay of organoids. Data presented as means ± SEM from three independent experiments. ***P < 0.001, two-way ANOVA. (n) Separated and merged images of immunofluorescent staining to detect Ki-67 (red), E-cadherin (green), DAPI (blue) in indicated organoids (scale bar, 20 μm)

To discover the association between STOML2 expression and malignant phenotype of CRC, STOML2 protein level was detected in seven human-derived CRC cell lines, among which SW620 presented the highest STOML2 expression and SW480 the lowest (Supplementary Fig. 2A). Therefore, we established STOML2-knockdown SW620 cell line with lentivirus transfection (−shSTOML2), and STOML2-overexpressed SW480 cell line with 3 × Flag-tagged STOML2 lentivirus (−STOML2), of which transfection efficiency was confirmed by immuno-blotting assay (Fig. 2B & F). Knockdown of STOML2 attenuated cell-cycle progression in SW480 (Fig. 2A). Inhibited cell growth rate (Fig. 2C), less colony formation (Fig. 2D and E), and impaired migration and invasion ability in transwell assay (Supplementary Fig. 2B & C) was observed in STOML2-knockdown groups, compared with negative control. In contrast, in STOML2-overexpressed SW480, cell fraction in S-phase was elevated (Fig. 2G), cell proliferation rate was significantly enhanced (Fig. 2H), more colonies were formed (Fig. 2I and J), migration and invasion ability was promoted in transwell assay (Supplementary Fig. 2D & E).

Furthermore, colon tumor was dissected from 16-week-old *Apc*^*Min/+*^ mice and tumor cells were isolated for 3D culture, which were confirmed by HE and anti-Cytokeratin IHC staining (Supplementary Fig. 2G). Tumor organoids were transfected with STOML2 overexpressing lenti-virus, which yielded substantially larger organoids measured by surface area, compared with negative control (Fig.2K and L). To better elucidate the functional changes, we conducted CCK8 assay, which showed enhanced proliferation in *Apc*^*Min/+*^-STOML2 organoids (Fig. 2M). Immunofluorescent (IF) staining for proliferation marker Ki-67 and epithelial marker E-cadherin using transfected organoids indicated significantly higher expression of Ki-67 in STOML2-overexpressed epithelial tumor cell (Fig. 2N). These results show that overexpressed STOML2 significantly enhances the proliferation of CRC cell in vitro.

### STOML2 promotes CRC growth and progression in vivo

**Fig. 3:**
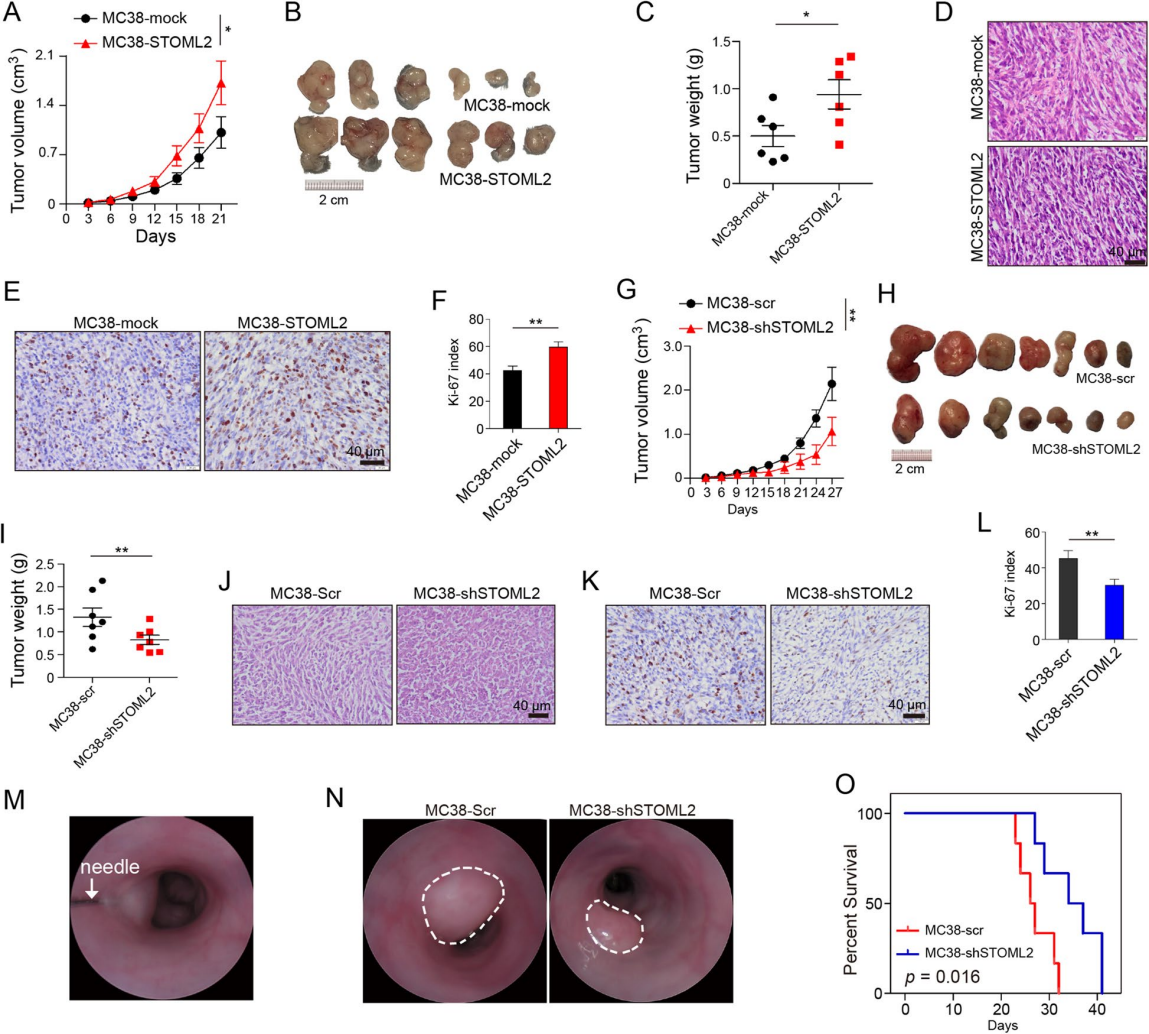
STOML2 promotes CRC growth and progression in vivo. **a**-**e** Subcutaneous implantation of STOML2-overexpressed MC38 cells (MC38-STOML2) and control group (MC38-mock) in C57BL/6 mice. (a) Tumor growth curves measured at indicated time points between MC38-STOML2 and -mock. *P < 0.05, two-way ANOVA. (b) 2 × 10^6^ cells were injected into hind limbs of each group; tumors were retrieved at 21st day after injection. (c) Tumor weight (g) measured at retrieval. *P < 0.05, *t* test. (d) Representative image of HE staining of subcutaneous implant in each group. Scale bar, 40 μm.. (e) Representative images of IHC staining show positive correlation between Ki-67 and STOML2 overexpression in subcutaneous implant in each group. Scale bar, 40 μm. (f) Relative intensity of Ki-67 staining, **P < 0.01, *t* test. **g**-**l** Subcutaneous implantation of STOML2-knockdown MC38 cells (MC38-shSTOML2) and control group (MC38-scr) in C57BL/6 mice. (g) Tumor volume (cm^3^) measured at indicated time point. **P < 0.01, two-way ANOVA. (h) 2 × 10^6^ cells were injected into hind limbs of each group; tumors were retrieved at 27th day after injection. (i) Tumor weight (g) measured at retrieval. **P < 0.01, *t* test. (j) Representative image of HE staining of subcutaneous implant in each group. Scale bar, 40 μm. (k) Representative images of IHC staining show negative correlation between Ki-67 and STOML2 knockdown in subcutaneous implant in each group. Scale bar, 40 μm. (l) Relative intensity of Ki-67 staining, **P < 0.01, *t* test. **m**-**o** CRC orthotopic injection of MC38-Scr and –shSTOML2 cells, murine endoscopy monitoring and survival curve. (m) 1 × 10^6^ cells of each group were injected into each C57BL/6 colon sub-mucosa under endoscopy. White arrow indicates needle. (n) Endoscopy monitoring of orthotopic tumor growth in each group. (o) Kaplan-Meier survival curve of mice in each group. *P* value was determined using log-rank test

To investigate the biological function of STOML2 in vivo, STOML2-overexpressed murine CRC cell strain, MC38-STOML2, was injected subcutaneously to mice strains C57BL/6. Tumor size were measured and growth curves were drawn, which showed that tumor in MC38-STOML2 group grew faster than those in corresponding negative control (Fig. 3A-C). The morphology of subcutaneous tumor was confirmed by HE staining (Fig. 3D), and there was no obvious change in cell morphology between groups, except for the relatively obvious intercellular space. IHC showed more staining of proliferation marker Ki-67 (Fig.3E & F), suggesting enhanced STOML2 expression markedly accelerated CRC cell proliferation in vivo*.* As for STOML2-knockdown cell line MC38-shSTOML2, comparing with negative control MC38-scr, tumor growth was significantly attenuated (Fig. 3G-I), and Ki-67 expression was lower (Fig. 3J-L). Similar findings were shown in subcutaneous implant model of STOML2-knockdown CT26 cell line in BALB/c mice (Supplementary Fig.2 H-M).

To simulate colorectal cancer formation, orthotopic model was established by inoculation of above mentioned MC38/CT26-scr and -shSTOML2 cells into colon lamina propria of syngeneic mice, under inspection of endoscopy (Fig. 3M). We found slower and smaller tumor growth in -shSTOML2 groups compared with -scr group (Fig. 3N). Moreover, mice implanted with -shSTOML2 cells had significant longer survival than those with control group cells (Fig.3O; log-rank *P =* 0.016). Thus, results above prove that STOML2 knockdown attenuates proliferation of CRC cells in vivo, both in subcutaneous and orthotopic tumor model.

### STOML2 interacts with PHB to modulate RAF/MEK/ERK MAPK pathway

**Fig. 4:**
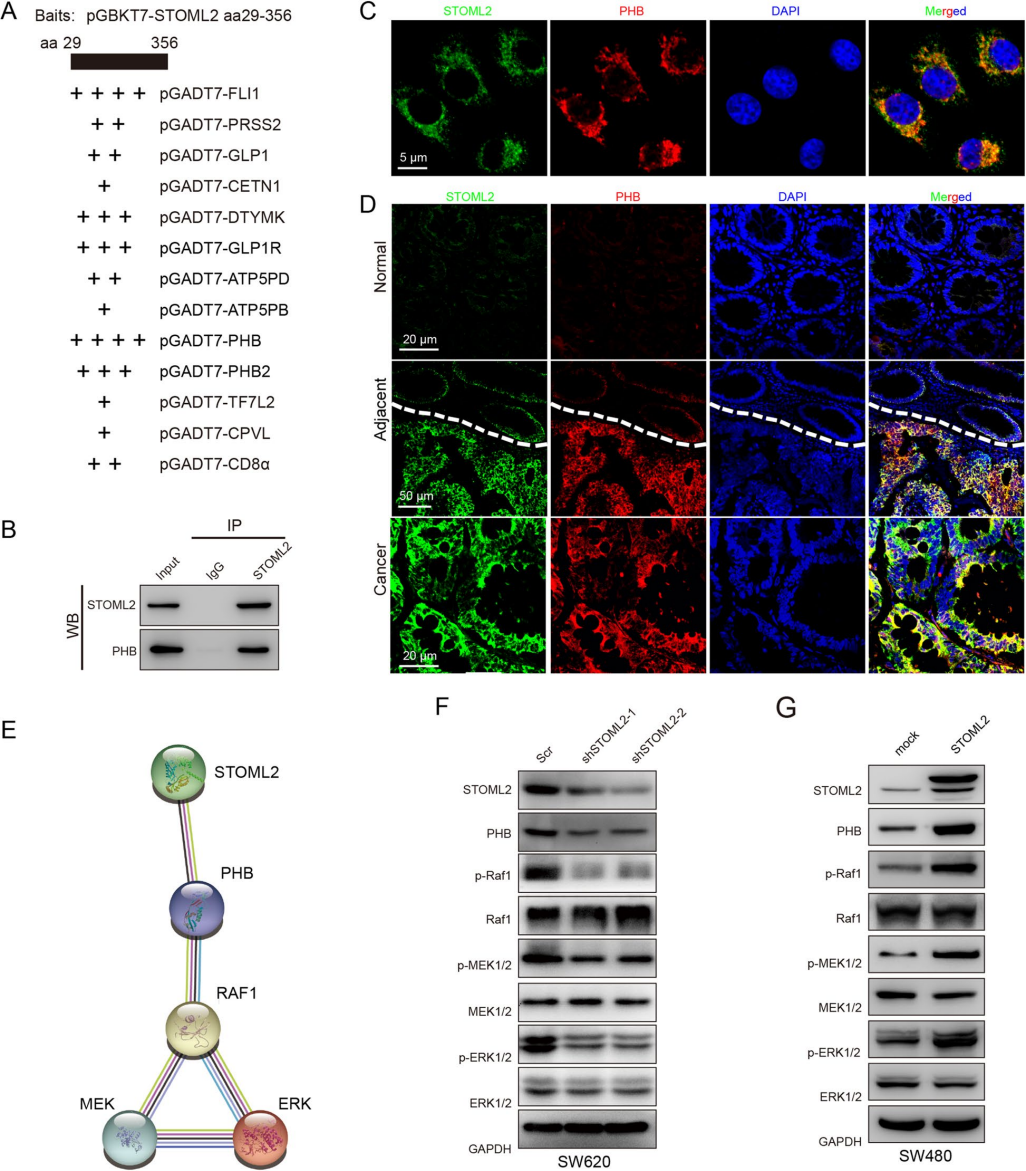
STOML2 interacts with PHB to modulate RAF/MEK/ERK MAPK pathway. **a** Yeast two-hybrid assay. Prey plasmids in 27 positive clones were extracted and sequenced. Sequences were looked up in PUBMED-BLAST for corresponding gene. “+” sign indicates counts of each gene from 29 positive clones. **b** Co-immunoprecipitation to validated STOML2 and DTYMK interaction in 293 T cells. **c** In vitro binding of STOML2 and PHB in co-IP analysis. **d** Immunostaining analysis of STOML2 (green), PHB (red) and DAPI (blue) in SW620-STOML2. Scale bar, 5 μm. **e** Formalin fixed paraffin embedded (FFPE) sections from CRC tumor-adjacent tissue were subjected to immunostaining analysis of STOML2 (green), PHB (red) and DAPI for DNA (blue). Scale bar, 20 μm (upper and lower row), 50 μm (middle row). **f** Association of STOML2, PHB, RAF1 and MAPK signaling pathway from STRING database. Interaction score: STOML2 and PHB (0.916); PHB and RAF1 (0.965); RAF1 and ERK (0.964); RAF1 and MEK (0.516); MEK and ERK (0.645). **g** Immunoblot analysis of STOML2, PHB and MAPK signaling pathway in SW620-scr and -shSTOML2 cells. **h** Immunoblot analysis of STOML2, PHB and MAPK signaling pathway in SW480-mock and –STOML2 cells

In order to clarify the regulatory mechanism of STOML2 in CRC, Y2H assay was used to screen the proteins binding to STOML2. The open reading frame fused to the GAL4-AD in plasmid of 29 positive clones was sequenced, and 13 corresponding proteins were identified, among which “PHB”, “PHB2”, “FLI1”, “DTYMK”, “GLP1R” were the most referred ones (Fig. 4A, Table 2). To further validate protein that most possibly interacts with STOML2, we performed GSEA of STOML2 and 13 candidates, using CRC sample expression profiling datasets GSE14333 (*n* = 290) [32] and GSE17538 (*n* = 238) [33–36] on GEO database (Supplementary Fig.3, 4, 5, 6). We picked top 20 pathway terms with highest normalized enrichment score (NES) of each gene; as is shown, overexpressed STOML2 was correlated with upregulation in Kyoto Encyclopedia of Genes and Genomes (KEGG) pathways like “RNA polymerase”, “pyrimidine metabolism”, “cell cycle”, “DNA replication”, indicating its functional connection with proliferation in CRC (Supplementary Fig.3A). In these top 20 terms, ranking of prey genes that most overlapped with STOML2 was: DTYMK (16 out of 20), PHB (14), PRSS2 (9), PHB2 (8), ATP5B (6), CD8a (2), TCF7L2 (2), GLP1 (1), GLP1R (1). Terms such as “cell cycle”, “pyrimidine metabolism”, “RNA polymerase”, “DNA replication”, “mismatch repair”, “homologous recombination”, were shared between DTYMK/PHB and STOML2 in KEGG pathways (Supplementary Fig.3& 6), whereas terms like “ribosome biogenesis”, “rRNA metabolic process”, “ribonucleoprotein complex subunit organization”, “ribonucleoprotein complex biogenesis”, “translational elongation” were shared in Gene Ontology biological process (GO-BP, Supplementary Fig.4A-C & 5A-C), which hinted that DTYMK and PHB were the most functionally similar proteins with STOML2.

**Table 2:** The results of yeast two-hybrid screening assay

Gene name	Official full name	Ensembl ID	Description	Positive count
PHB	Prohibitin	00000167085	Regulates mitochondrial respiration activity and in aging.	4
FLI1	Fli-1 proto-oncogene, ETS transcription factor	00000151702	Sequence-specific transcriptional activator recognizes the DNA sequence 5′-C [CA]GGAAGT-3′.	4
PHB2	Prohibitin 2	00000215021	Mediator of transcriptional repression by nuclear hormone receptors via recruitment of histone deacetylases.	3
DTYMK	Deoxythymidylate Kinase	00000168393	Catalyzes the conversion of dTMP to dTDP.	3
GLP1R	Glucagon Like Peptide 1 Receptor	00000112164	Signal activation of adenylyl cyclase and increased intracellular cAMP levels.	3
ATP5PD	ATP Synthase Peripheral Stalk Subunit D	00000167863	Proton transmembrane transporter activity.	2
PRSS2	Serine protease 2	00000275896	Expressed in pancreas and involved in defensin processing in ileum.	2
GLP1	Glucagon	00000115263	Regulates glucose metabolism and homeostasis	2
CD8A	T-cell surface glycoprotein CD8 alpha chain isoform 2 precursor	00000153563	Coreceptor for MHC class I molecule: peptide complex In T-cells.	2
CETN1	Centrin 1	00000177143	microtubule-organizing center structure and function	1
ATP5PB	ATP synthase peripheral stalk-membrane subunit b	00000167863		1
CPVL	Carboxypeptidase Vitellogenic Like	00000106066	Digestion of phagocytosed particles in the lysosome, inflammatory protease cascade, and trimming of peptides for antigen presentation.	1
TCF7L2	Transcription Factor 7 Like 2	00000148737	Participates in the Wnt signaling pathway and modulates MYC expression by binding to its promoter in a sequence-specific manner	1

However, binding between STOML2 and DTYMK could not be verified by co-immunoprecipitation (co-IP; Fig. 4B). Since DTYMK was excluded, we turned our focus to PHB. Co-IP confirmed binding interaction between STOML2 and PHB in vitro (Fig. 4C). IF staining in SW620 cell line indicated co-localization of STOML2 and PHB in cytoplasm (Fig. 4D). In comparison of tissue sections from our biobank, IF staining demonstrated low STOML2 and PHB expression in normal colon, and evident co-localization between STOML2 and PHB in CRC lesions, with clear margin at the invasive front in tumor-adjacent tissue (Fig.4E). STRING database [37] also suggested PHB as functional partner of STOML2 with high score (0.916; Fig. 4F), which indeed had been identified to play indispensable role in the activation of the RAF-MEK-ERK pathway by RAS [38]. In STOML2-knockdown SW620, PHB protein expression decreased simultaneously, accompanied by decreased phosphorylation of mitogen-activated protein kinase (MAPK) pathway proteins, such as RAF, MEK and ERK (Fig.4G); whereas opposite effect took place on PHB and phosphorylation of MAPK pathway proteins, in STOML2-overexpressed SW480 cells (Fig.4H).

### PHB-knockdown impairs STOML2-induced CRC proliferation and tumor growth

**Fig. 5:**
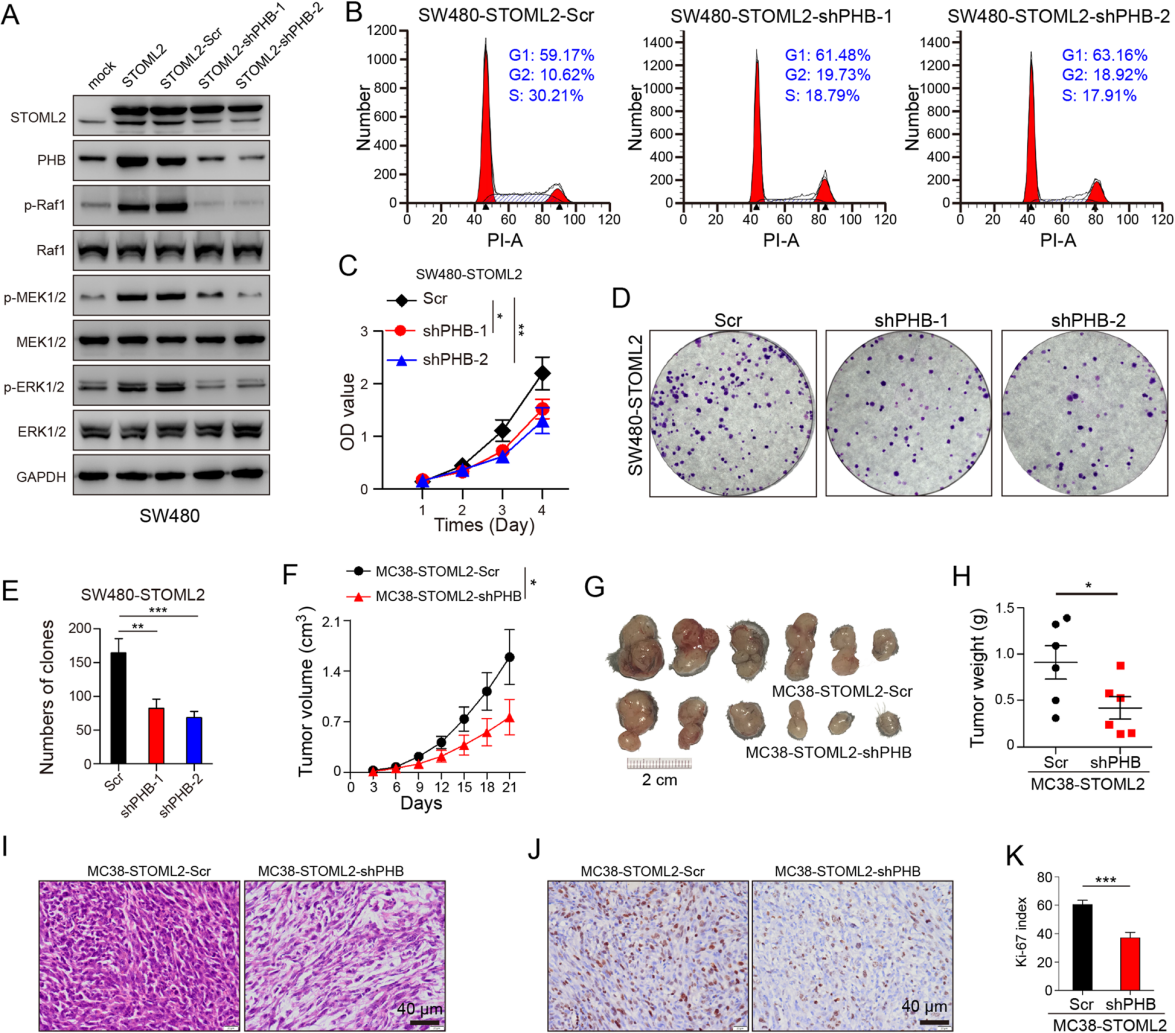
PHB-knockdown impairs STOML2-induced CRC proliferation and tumor growth. **a** Immunoblot analysis of STOML2, PHB and MAPK signaling pathway in SW480-STOML2 cells, with/without PHB knockdown. **b** Cell-cycle distribution analysis of STOML2-overexpressed PHB-knockdown SW480 and control. Fraction of cells in G1/G2/S phase was shown. **c** CCK8 proliferation assay. Indicated cells were seeded 5000/well in 96-well culture-plates and OD values were measured by day. Data are presented as means ± SEM from five independent experiments. *P < 0.05, **P < 0.01, two-way ANOVA. **d** Colony formation of indicated cells. 5000 indicated cells were seeded in 6 wells culture-plates. Giemsa staining was carried out after 14 days of culture. **e** Number of colonies formed on each plate. Data presented as means ± SEM from three independent experiments. ***P < 0.001, **P < 0.01, one-way ANOVA. **f-k** Subcutaneous implantation of MC38-STOML2-scr and MC38-STOML2-shPHB cells in C57BL/6 mice. (f) Tumor volume (cm^3^) measured at indicated time points between two groups. *P < 0.05, two-way ANOVA. (g) 2 × 10^6^ cells were injected into hind limbs of each group; tumors were retrieved at 21st day after injection. (h) Tumor weight (*g*) measured at retrieval. **P < 0.01, *t* test. (i) Representative image of HE staining of subcutaneous implant in each group. Scale bar, 40 μm. (j) Representative images of IHC staining show negative correlation between Ki-67 and PHB knockdown in each group. Scale bar, 40 μm. (k) Relative intensity of Ki-67 staining, ***P < 0.001, *t* test

To assess the role of PHB in STOML2-induced CRC oncogenesis, we knocked down PHB in STOML2-overexpressed SW480 cells. STOML2 overexpression was accompanied by upregulation of PHB and phosphorylation of RAF, MEK and ERK, which could be diminished by knockdown of PHB (Fig.5A). To better elucidate functional changes in SW480-STOML2 + shPHB cells, cell cycle analysis, CCK8 and colony formation assay were conducted, which revealed that inhibition of PHB was capable of impairing STOML2-induced cell proliferation (Fig.5B & C) and colony formation in SW480 in vitro (Fig.5D & E). Subcutaneous implantation of MC38-STOML2-shPHB cells showed slower and smaller tumor growth, compared with STOML2-overexpressed counterpart (Fig.5F-H). The morphology of subcutaneous tumor was confirmed by HE staining (Fig. 5I), and less staining of Ki-67 was found in MC38-STOML2-shPHB tumors (Fig. 5J & K). These results indicated that PHB-knockdown markedly impaired STOML2-induced CRC cell proliferation, both in vitro and in vivo.

### RAF1 inhibitor sorafenib attenuates STOML2-induced CRC proliferation and tumor growth

**Fig. 6:**
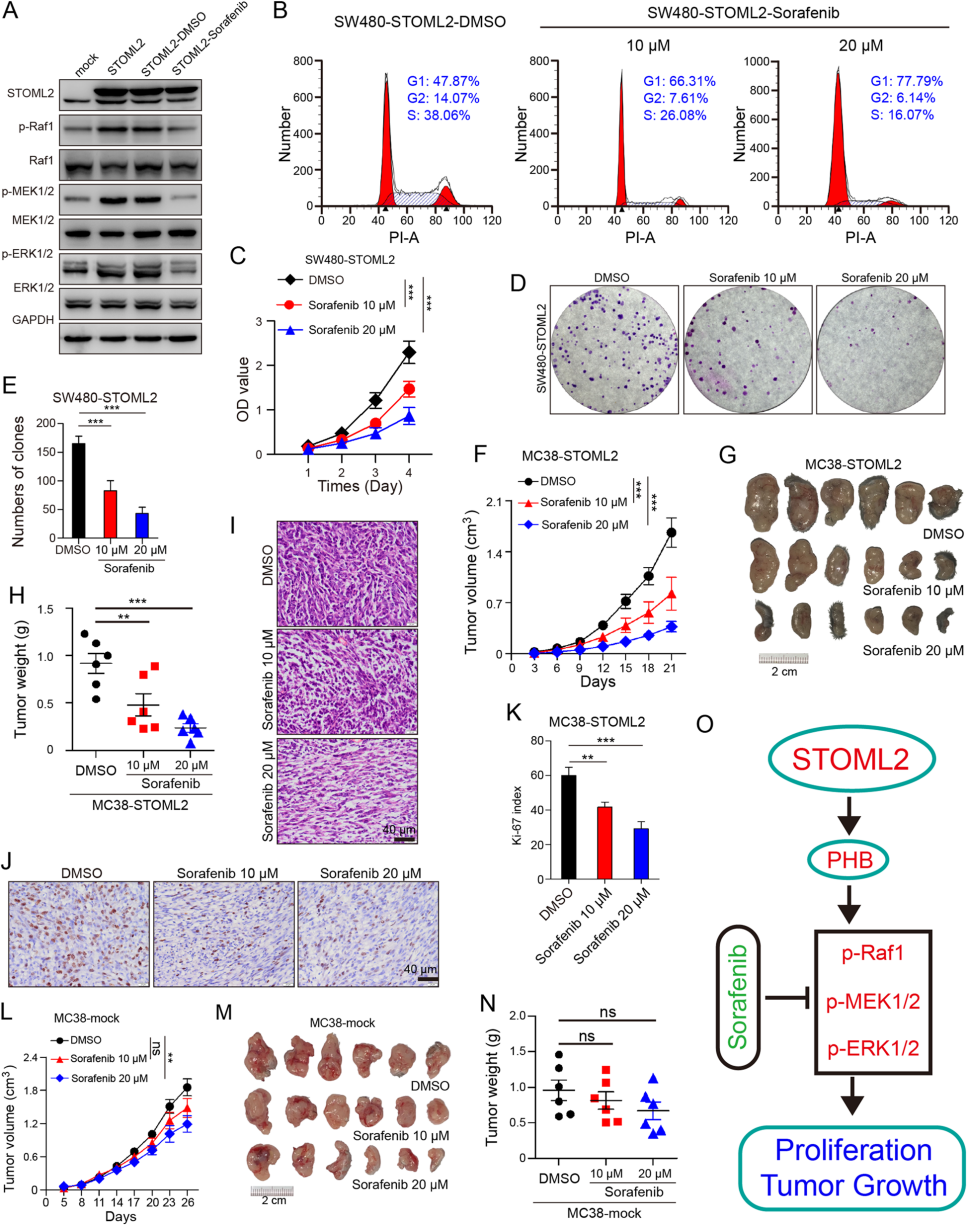
RAF1 inhibitor sorafenib attenuates STOML2-induced CRC proliferation and tumor growth. **a** Immunoblot analysis of STOML2, PHB and MAPK signaling pathway in SW480-STOML2 cells, after administration of sorafenib (10 μM, 24 h) or DMSO. **b** Cell-cycle distribution analysis of STOML2-overexpressed SW480 and control after sorafenib (10 or 20 μM) or DMSO administration. Fraction of cells in G1/G2/S phase was shown. **c** CCK8 proliferation assay. SW480-STOML2 cells were seeded 5000 per well in 96-well culture plate; after administration of DMSO or sorafenib (10 or 20 μm), OD values were measured by day. Data are presented as means ± SEM from five independent experiments. ***P < 0.001, two-way ANOVA. **d** Colony formation of SW480-STOML2 cells. 5000/well of indicated cells were seeded in 6-well culture-plates. DMSO or 10, 20 μM sorafenib was administrated before Giemsa staining at 14th day culture. **e** Number of colonies formed on each plate. Data presented as means ± SEM from three independent experiments. ***P < 0.001, one-way ANOVA. **f-n** Subcutaneous implantation of MC38-STOML2 and MC38-mock cells in C57BL/6 mice with administration of sorafenib (10 or 20 μM) or DMSO. (f) Subcutaneous implantation of DMSO or 10, 20 μM sorafenib treated MC38-STOML2 cells in C57BL/6 mice. Tumor volume (cm^3^) measured at indicated time points. ***P < 0.001, two-way ANOVA. (g) 2 × 10^6^ cells were injected into hind limbs of each group; tumors were retrieved at 21st day after injection. (h) Tumor weight (*g*) measured at retrieval. **P < 0.01, ***P < 0.001, *t* test. (i) Representative image of HE staining of subcutaneous implant in each group. Scale bar, 40 μm. (j) Representative images of IHC staining show negative correlation between Ki-67 and sorafenib dosage in each group. Scale bar, 40 μm. (k) Relative intensity of Ki-67 staining. **P < 0.01, ***P < 0.001, *t* test. (l) Subcutaneous implantation of DMSO or 10, 20 μM sorafenib treated MC38-STOML2 cells in C57BL/6 mice. Tumor volume (cm^3^) measured at indicated time points. **P < 0.01, ns, not significant, two-way ANOVA. (m) 2 × 10^6^ cells were injected into hind limbs of each group; tumors were retrieved at 26th day after injection. (n) Tumor weight (*g*) measured at retrieval. ns, not significant, *t* test. **o** Schematic depiction of regulatory mechanism underlying CRC proliferation via STOML2/PHB/MAPK signaling and therapeutic target

To verify therapeutic value of STOML2 in MAPK pathway, experimental therapy targeting MAPK pathway was conducted in STOML2-overexpressed cells, which were treated with RAF1 inhibitor sorafenib. Sorafenib inhibited STOML2-induced activation of MAPK pathway (Fig. 6A), which significantly attenuated cell cycle progression (Fig. 6B), proliferation (Fig. 6C), colony formation (Fig. 6D & E) and migration/invasion ability in transwell assay (Supplementary Fig. 7A-D), in dose-dependent manner. Administration of sorafenib to MC38 subcutaneous tumors also presented impaired tumor growth (Fig. 6F) and size (Fig. 6G & H) in dose-dependent manner, as determined by Ki-67 staining (Fig. 6I-K), whereas sorafenib show no significant effect in negative control tumors (Fig. 6L-N), demonstrating its specific curative effect to STOML2-overexpressed CRC tumors in vivo.

Results above point out that STOML2 interacts with PHB, promotes CRC proliferation by regulating MAPK pathway; disrupting STOML2-PHB interaction and subsequently activated MAPK signaling pathway is proved to be promising therapeutic strategy for STOML2-overexpressed CRC (Fig. 6O).

## Discussion

Cancer cell is characterized by its ability of infinite proliferation, under influence of persistent activation or inhibition of signaling pathways, triggered by alteration on expression level of certain genes affecting these pathways [39, 40]. In the present study, STOML2 is the key oncogene screened out from medical center biobank by our research team, which is highly expressed, and related to tumorigenesis, progression and prognosis of gastroenteric cancers.

From precedent researches, we have revealed the elevation of STOML2 in gastric cancer on tissue and molecular level, and its correlation with various clinical indicators, which also verified elevation of STOML2 as an independent prognostic factor for gastric cancer, provided first and valid evidence to explore the specific molecular mechanism of it in malignant proliferation of gastric cancer [21]. In this study, an increase in STOML2 expression in CRC is discovered and its significant association with adverse clinical factors of CRC patients is confirmed, which indicates that STOML2-upregulated CRC exhibits malignant proliferating phenotype such as proliferation, cell cycle, invasion and metastasis. This research was intended initially to further explore novel interacting proteins of STOML2 by Y2H assay; however, similar STOML2-PHB interaction was found and verified with co-IP. Combining our work of STOML2 in both GC and CRC, it may be an important research direction to further explore the interaction domain of STOML2 and PHB, and search for blocking agent.

The biological role of STOML2 is to regulate mitochondrial membrane stability and function; under mitochondrial stress, it interacts with prohibitins, molecular chaperones in respiratory chain complexes [7]. Christie et al. demonstrated direct binding by STOML2 (referred to as SLP-2) to PHB-1, which our finding concurs with, and PHB-driven mitogenesis could partially explain why STOML2 could apparently regulate proliferation [41]. Hu et al. reported that STOML2 activated MEK/ERK signaling and suppressed mitochondrial apoptosis pathway in HeLa cervical cancer cells, through altering the ability of mitochondria to buffer Ca^2+^ and shape cytosolic Ca^2+^ signaling [42]. Recent related studies also implied that, STOML2 modulated tumor malignancy via IL6-STAT3 pathway in head and neck squamous cell carcinoma [19]. In the study conducted by Zhu et al., silence of STOML2 repressed migration and invasion ability of liver cancer via inhibiting the NF-κB Pathway [18]. Zhou et al. investigated STOML2 expression in CRC and its association with patient prognosis (*n* = 95; follow-up period = 60 months), detected the variations of canonical Wnt/β-catenin signaling pathway after STOML2 inhibition in CRC cells to enhance cell growth [43]; in comparison, our work provided a larger patient cohort (*n* = 215) and longer follow-up period (120 months) from our center, and discovered STOML2 and PHB interaction in CRC regulating MAPK signaling. Based on the results of current study and evidence from other researchers, we suppose that similar effect occurs in STOML2-elevated CRC cells, through which promotes cancer cell proliferation, and is worth further evaluation. These accumulating evidence indicates that STOML2 broadly participates in various signaling network, and serves as an important pro-tumorigenic gene in CRC.

In this study, novel techniques were utilized in the exploration of malignant mechanism of STOML2 in CRC, which provided more convincing evidence. Organoid culture along with subsequent IF staining provides near-physiologic cellular composition and behavior, which permits study of morphology during tumor formation. Orthotopic model combined with routine endoscopic monitoring provides several advantages over traditional subcutaneous implantation. Orthotopic injection furnishes better simulation to micro-environment for CRC carcinogenesis, while mouse endoscopy is conducive to continuously monitoring tumor burden in vivo without causing damage, and reduces the need for multiple cohorts of experimental mice, in terms of animal welfare.

The pivotal role of STOML2 in CRC progression was validated through current research. For proteins which are overexpressed and links with malignancy in various cancers, multiple perspectives could be targeted in terms of therapeutic strategy, e.g. antibody or recombinant protein treatment for membrane proteins [44, 45], and small molecule drugs targeting downstream pathway of intracellular proteins. In this study, based on subcellular localization of STOML2 in cytoplasm, Y2H assay combining bioinformatic analysis was implemented to screen out putative interacting proteins of STOML2, with which STOML2 shares similar biological function and regulation of signaling pathways. Among 13 putative candidates, we started from DTYMK and PHB with the highest rank, confirmed interaction between STOML2 and PHB, which also presented significant co-localization in cell line and tissue. Other candidates from Y2H assay also showed promising research value. For instance, since the major pool of STOML2 is associated with mitochondria, ATP synthases like ATP5B and ATP5H also share resemblance with STOML2 in function and subcellular localization. It was reported that STOML2 is an important player in T cell activation by ensuring sustained TCR signaling, which could be connected with CD8A [46–48].

Sorafenib is a multi-target kinase inhibitor that blocks cell proliferation by inhibiting the MAPK pathway, and prevents tumor-associated angiogenesis by inhibiting vascular endothelial growth factor receptors (VEGFR) as well as platelet-derived growth factor receptor (PDGFR) and FLT3 [49–51]. It has been approved for the treatment of patients with unresectable hepatocellular carcinoma and advanced renal cell carcinoma (http://www.brimr.org/PKI/PKIs.htm). Studies on clinical application of sorafenib in CRC patients have been conducted in recent decades [51, 52], and our findings have validated that sorafenib attenuates STOML2-induced CRC proliferation and tumor growth, with antagonizing effect to MAPK pathway (Fig. 6A), which may provide evidence for potential use of sorafenib in decreasing tumor burden of CRC. However, the introduction of sorafenib to CRC treatment needs to proceed with caution, due to its multi-targeting effect and non-specificity to MAPK pathway. Furthermore, we shall proceed to in-depth investigation on binding site and interacting domain between STOML2 and PHB, in order to be more pertinent in treatment study and to develop more specific inhibitors.

## Conclusions

This study presents that STOML2 is significantly upregulated in CRC and promotes cancer cell proliferation and tumor growth through MAPK signaling pathway by interaction with PHB, which suggests STOML2 as novel, potent biomarker and therapeutic target in CRC, urging for further investigation.

## Supplementary Information

**Additional file 1.**

**Additional file 2.**

**Additional file 3.**

**Additional file 4.**

**Additional file 5.**

**Additional file 6.**

**Additional file 7.**

**Additional file 8.**

**Additional file 9.**

## Data Availability

The datasets generated during and/or analysed during the current study (GSE14333 and GSE17538) are available in Gene Expression Omnibus (GEO) database, “https://www.ncbi.nlm.nih.gov/geo/query/acc.cgi?acc=GSE14333” [32] and “https://www.ncbi.nlm.nih.gov/geo/query/acc.cgi?acc=GSE17538” [33–36].

## Abbreviations

CRC: Colorectal cancer
STOML2: Stomatin-like 2
CCK8: Cell counting kit 8
PMSF: Phenylmethylsulfonyl fluoride
PBS: Phosphate Buffered Saline
SDS-PAGE: Sodium dodecyl sulfatepolyacrylamide gel electrophoresis
PVDF: Poly (vinylidene fluoride)
FFPE: Formalin fixed paraffin-embedded
HRP: Horseradish Peroxidase
SD: Synthetic dropout medium
DDO: Double dropout supplement
QDO: Quadruple dropout supplement
SEM: Standard error of mean
GSEA: Gene set enrichment analysis
GEO: Gene expression omnibus
PHB: Prohibitin
DTYMK: Deoxythymidylate kinase
MAPK: Mitogen-activated protein kinase

## References

[1] Bray F, Ferlay J, Soerjomataram I, Siegel RL, Torre LA, Jemal A (2018). Global cancer statistics 2018: GLOBOCAN estimates of incidence and mortality worldwide for 36 cancers in 185 countries. CA Cancer J Clin.

[2] Torre LA, Bray F, Siegel RL, Ferlay J, Lortet-Tieulent J, Jemal A (2015). Global cancer statistics, 2012. CA Cancer J Clin.

[3] Allemani C, Matsuda T, Di Carlo V, Harewood R, Matz M, Nikšić M (2018). Global surveillance of trends in cancer survival 2000–14 (CONCORD-3): analysis of individual records for 37 513 025 patients diagnosed with one of 18 cancers from 322 population-based registries in 71 countries. Lancet.

[4] Keum N, Giovannucci E (2019). Global burden of colorectal cancer: emerging trends, risk factors and prevention strategies. Nat Rev Gastroenterol Hepatol.

[5] Dekker E, Tanis PJ, Vleugels JLA, Kasi PM, Wallace MB (2019). Colorectal cancer. Lancet.

[6] Wang Y, Morrow JS (2000). Identification and characterization of human SLP-2, a novel homologue of stomatin (band 7.2b) present in erythrocytes and other tissues. J Biol Chem.

[7] Wang Y, Cao W, Yu Z, Liu Z (2009). Downregulation of a mitochondria associated protein SLP-2 inhibits tumor cell motility, proliferation and enhances cell sensitivity to chemotherapeutic reagents. Cancer Biol Ther.

[8] Hajek P, Chomyn A, Attardi G (2007). Identification of a novel mitochondrial complex containing mitofusin 2 and stomatin-like protein 2. J Biol Chem.

[9] Zhang L, Ding F, Cao W, Liu Z, Liu W, Yu Z (2006). Stomatin-like protein 2 is overexpressed in cancer and involved in regulating cell growth and cell adhesion in human esophageal squamous cell carcinoma. Clin Cancer Res.

[10] Cui Z, Zhang L, Hua Z, Cao W, Feng W, Liu Z (2007). Stomatin-like protein 2 is overexpressed and related to cell growth in human endometrial adenocarcinoma. Oncol Rep.

[11] Song L, Liu L, Wu Z, Lin C, Dai T, Yu C (2012). Knockdown of stomatin-like protein 2 (STOML2) reduces the invasive ability of glioma cells through inhibition of the NF-kappaB/MMP-9 pathway. J Pathol.

[12] Liu Z, Yang Y, Zhang Y, Ye X, Wang L, Xu G (2014). Stomatin-like protein 2 is associated with the clinicopathological features of human papillary thyroid cancer and is regulated by TGF-beta in thyroid cancer cells. Oncol Rep.

[13] Xiao B, Xie Z, Guo L, Wu J, Zhang H (2015). Stomatin-like protein 2 expression is associated with clinical survival in patients with cervical cancer. Int J Clin Exp Pathol.

[14] Sun F, Ding W, He JH, Wang XJ, Ma ZB, Li YF (2015). Stomatin-like protein 2 is overexpressed in epithelial ovarian cancer and predicts poor patient survival. BMC Cancer.

[15] Guo XY, Guo HF, Guo HM (2020). Clinical significance of SLP-2 in epithelial ovarian cancer and its regulatory effect on the notch signaling pathway. Eur Rev Med Pharmacol Sci.

[16] Zhang L, Liu FJ (2017). Expression of SLP-2 gene and CCBE1 are associated with prognosis of rectal cancer. Eur Rev Med Pharmacol Sci.

[17] Liu Q, Li A, Wang L, He W, Zhao L, Wu C (2020). Stomatin-like protein 2 promotes tumor cell survival by activating the JAK2-STAT3-PIM1 pathway, suggesting a novel therapy in CRC. Mol Ther Oncolytics.

[18] Zhu W, Li W, Geng Q, Wang X, Sun W, Jiang H (2018). Silence of Stomatin-like protein 2 represses migration and invasion ability of human liver Cancer cells via inhibiting the nuclear factor kappa B (NF-kappaB) pathway. Med Sci Monit.

[19] Qu H, Jiang W, Wang Y, Chen P (2019). STOML2 as a novel prognostic biomarker modulates cell proliferation, motility and chemo-sensitivity via IL6-Stat3 pathway in head and neck squamous cell carcinoma. Am J Transl Res.

[20] Liu D, Zhang L, Shen Z, Tan F, Hu Y, Yu J (2013). Increased levels of SLP-2 correlate with poor prognosis in gastric cancer. Gastric Cancer.

[21] Ma W, Xu Z, Wang Y, Li W, Wei Z, Chen T (2018). A positive feedback loop of SLP2 activates MAPK signaling pathway to promote gastric Cancer progression. Theranostics..

[22] Network CGA (2012). Comprehensive molecular characterization of human colon and rectal cancer. Nature..

[23] Cho EJ, Kim M, Jo D, Kim J, Oh JH, Chung HC (2021). Immuno-genomic classification of colorectal cancer organoids reveals cancer cells with intrinsic immunogenic properties associated with patient survival. J Exp Clin Cancer Res.

[24] Lappano R, Talia M, Cirillo F, Rigiracciolo DC, Scordamaglia D, Guzzi R (2020). The IL1beta-IL1R signaling is involved in the stimulatory effects triggered by hypoxia in breast cancer cells and cancer-associated fibroblasts (CAFs). J Exp Clin Cancer Res.

[25] Xue X, Shah YM (2013). In vitro organoid culture of primary mouse colon tumors. J Vis Exp.

[26] Schwank G, Koo BK, Sasselli V, Dekkers JF, Heo I, Demircan T (2013). Functional repair of CFTR by CRISPR/Cas9 in intestinal stem cell organoids of cystic fibrosis patients. Cell Stem Cell.

[27] Dow LE, O'Rourke KP, Simon J, Tschaharganeh DF, van Es JH, Clevers H (2015). Apc restoration promotes cellular differentiation and reestablishes crypt homeostasis in colorectal Cancer. Cell..

[28] Ernst M, Preaudet A, Putoczki T. Non-invasive assessment of the efficacy of new therapeutics for intestinal pathologies using serial endoscopic imaging of live mice. J Vis Exp. 2015;97. 10.3791/52383 PMC4401233 25867916

[29] Subramanian A, Tamayo P, Mootha VK, Mukherjee S, Ebert BL, Gillette MA (2005). Gene set enrichment analysis: a knowledge-based approach for interpreting genome-wide expression profiles. Proc Natl Acad Sci U S A.

[30] Mootha VK, Lindgren CM, Eriksson KF, Subramanian A, Sihag S, Lehar J (2003). PGC-1alpha-responsive genes involved in oxidative phosphorylation are coordinately downregulated in human diabetes. Nat Genet.

[31] Wu C, Orozco C, Boyer J, Leglise M, Goodale J, Batalov S (2009). BioGPS: an extensible and customizable portal for querying and organizing gene annotation resources. Genome Biol.

[32] Jorissen RN, Gibbs P, Christie M, Prakash S, Lipton L, Desai J (2009). Metastasis-associated gene expression changes predict poor outcomes in patients with dukes stage B and C colorectal Cancer. Clin Cancer Res.

[33] Smith JJ, Deane NG, Wu F, Merchant NB, Zhang B, Jiang A (2010). Experimentally derived metastasis gene expression profile predicts recurrence and death in patients with colon cancer. Gastroenterology..

[34] Freeman TJ, Smith JJ, Chen X, Washington MK, Roland JT, Means AL (2012). Smad4-mediated signaling inhibits intestinal neoplasia by inhibiting expression of beta-catenin. Gastroenterology..

[35] Chen MS, Lo YH, Chen X, Williams CS, Donnelly JM, Criss ZK (2019). Growth factor-independent 1 is a tumor suppressor gene in colorectal Cancer. Mol Cancer Res.

[36] Williams CS, Bernard JK, Demory Beckler M, Almohazey D, Washington MK, Smith JJ (2015). ERBB4 is over-expressed in human colon cancer and enhances cellular transformation. Carcinogenesis..

[37] Szklarczyk D, Gable AL, Lyon D, Junge A, Wyder S, Huerta-Cepas J (2019). STRING v11: protein-protein association networks with increased coverage, supporting functional discovery in genome-wide experimental datasets. Nucleic Acids Res.

[38] Rajalingam K, Wunder C, Brinkmann V, Churin Y, Hekman M, Sievers C (2005). Prohibitin is required for Ras-induced Raf-MEK-ERK activation and epithelial cell migration. Nat Cell Biol.

[39] Wan PT, Garnett MJ, Roe SM, Lee S, Niculescu-Duvaz D, Good VM (2004). Mechanism of activation of the RAF-ERK signaling pathway by oncogenic mutations of B-RAF. Cell..

[40] Barker N, Clevers H (2006). Mining the Wnt pathway for cancer therapeutics. Nat Rev Drug Discov.

[41] Christie DA, Lemke CD, Elias IM, Chau LA, Kirchhof MG, Li B (2011). Stomatin-like protein 2 binds cardiolipin and regulates mitochondrial biogenesis and function. Mol Cell Biol.

[42] Hu G, Zhang J, Xu F, Deng H, Zhang W, Kang S (2018). Stomatin-like protein 2 inhibits cisplatin-induced apoptosis through MEK/ERK signaling and the mitochondrial apoptosis pathway in cervical cancer cells. Cancer Sci.

[43] Zhou C, Li Y, Wang G, Niu W, Zhang J, Wang G (2019). Enhanced SLP-2 promotes invasion and metastasis by regulating Wnt/beta-catenin signal pathway in colorectal cancer and predicts poor prognosis. Pathol Res Pract.

[44] Davenport AP, Scully CCG, de Graaf C, Brown AJH, Maguire JJ (2020). Advances in therapeutic peptides targeting G protein-coupled receptors. Nat Rev Drug Discov.

[45] Li E, Huang X, Zhang G, Liang T (2021). Combinational blockade of MET and PD-L1 improves pancreatic cancer immunotherapeutic efficacy. J Exp Clin Cancer Res.

[46] Christie DA, Kirchhof MG, Vardhana S, Dustin ML, Madrenas J (2012). Mitochondrial and plasma membrane pools of stomatin-like protein 2 coalesce at the immunological synapse during T cell activation. PLoS One.

[47] Kirchhof MG, Chau LA, Lemke CD, Vardhana S, Darlington PJ, Marquez ME (2008). Modulation of T cell activation by stomatin-like protein 2. J Immunol.

[48] Christie DA, Mitsopoulos P, Blagih J, Dunn SD, St-Pierre J, Jones RG (2012). Stomatin-like protein 2 deficiency in T cells is associated with altered mitochondrial respiration and defective CD4+ T cell responses. J Immunol.

[49] Lierman E, Lahortiga I, Van Miegroet H, Mentens N, Marynen P, Cools J (2007). The ability of sorafenib to inhibit oncogenic PDGFRbeta and FLT3 mutants and overcome resistance to other small molecule inhibitors. Haematologica..

[50] Abdelgalil AA, Alkahtani HM, Al-Jenoobi FI (2019). Sorafenib. Profiles Drug Subst Excip Relat Methodol.

[51] Roskoski R (2018). Targeting oncogenic Raf protein-serine/threonine kinases in human cancers. Pharmacol Res.

[52] Kircher SM, Nimeiri HS, Benson AB (2016). Targeting angiogenesis in colorectal Cancer: tyrosine kinase inhibitors. Cancer J.

