# Supplementary figures and images for: STOML2 interacts with PHB through activating MAPK signaling pathway to promote colorectal Cancer proliferation
Source: J Exp Clin Cancer Res. 2021 Nov 15;40:359. doi: 10.1186/s13046-021-02116-0 (PMC8591804; doi:10.1186/s13046-021-02116-0)

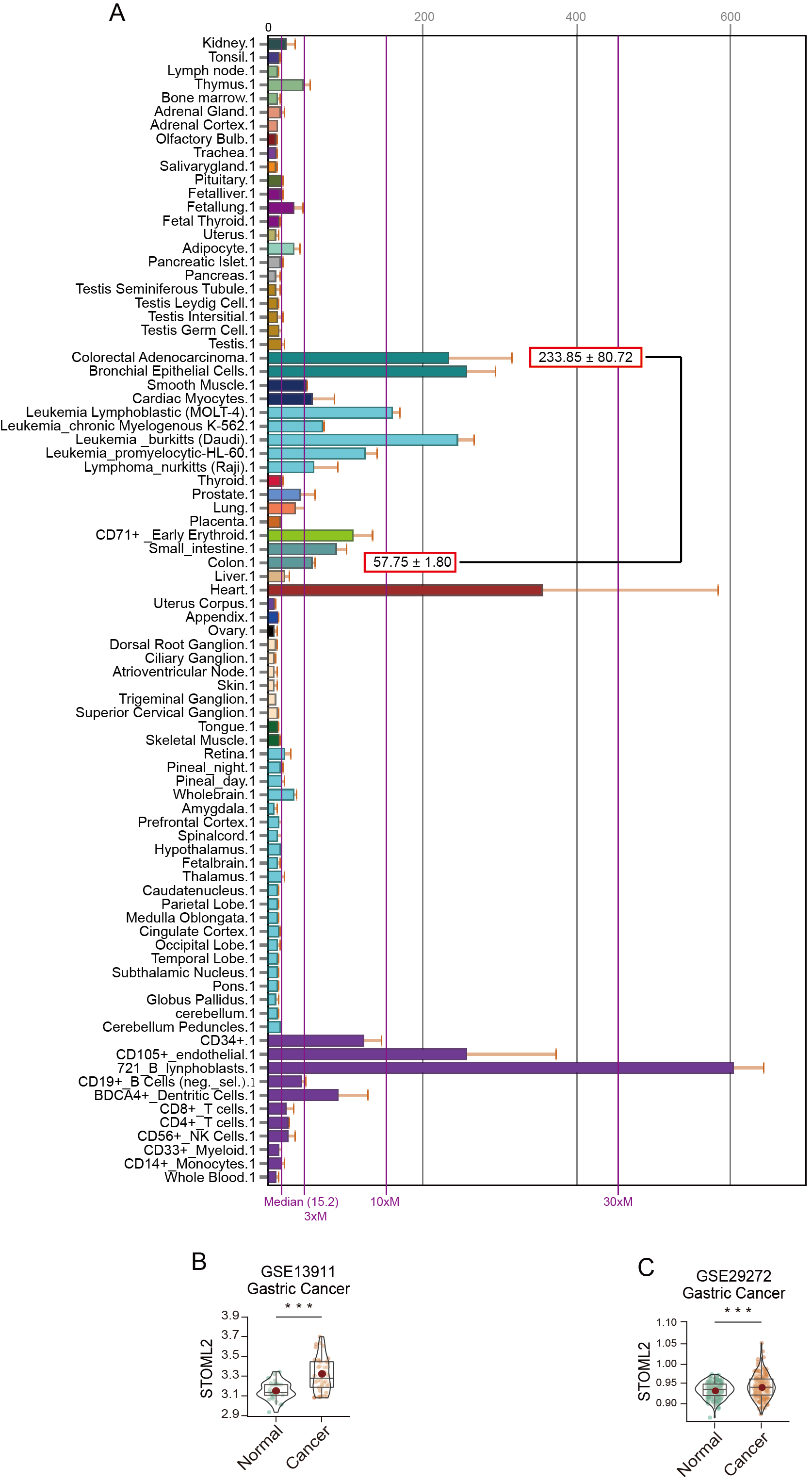

Supplement: Supplementary file 1 — Additional file 1. [file 13046_2021_2116_MOESM1_ESM.tif]

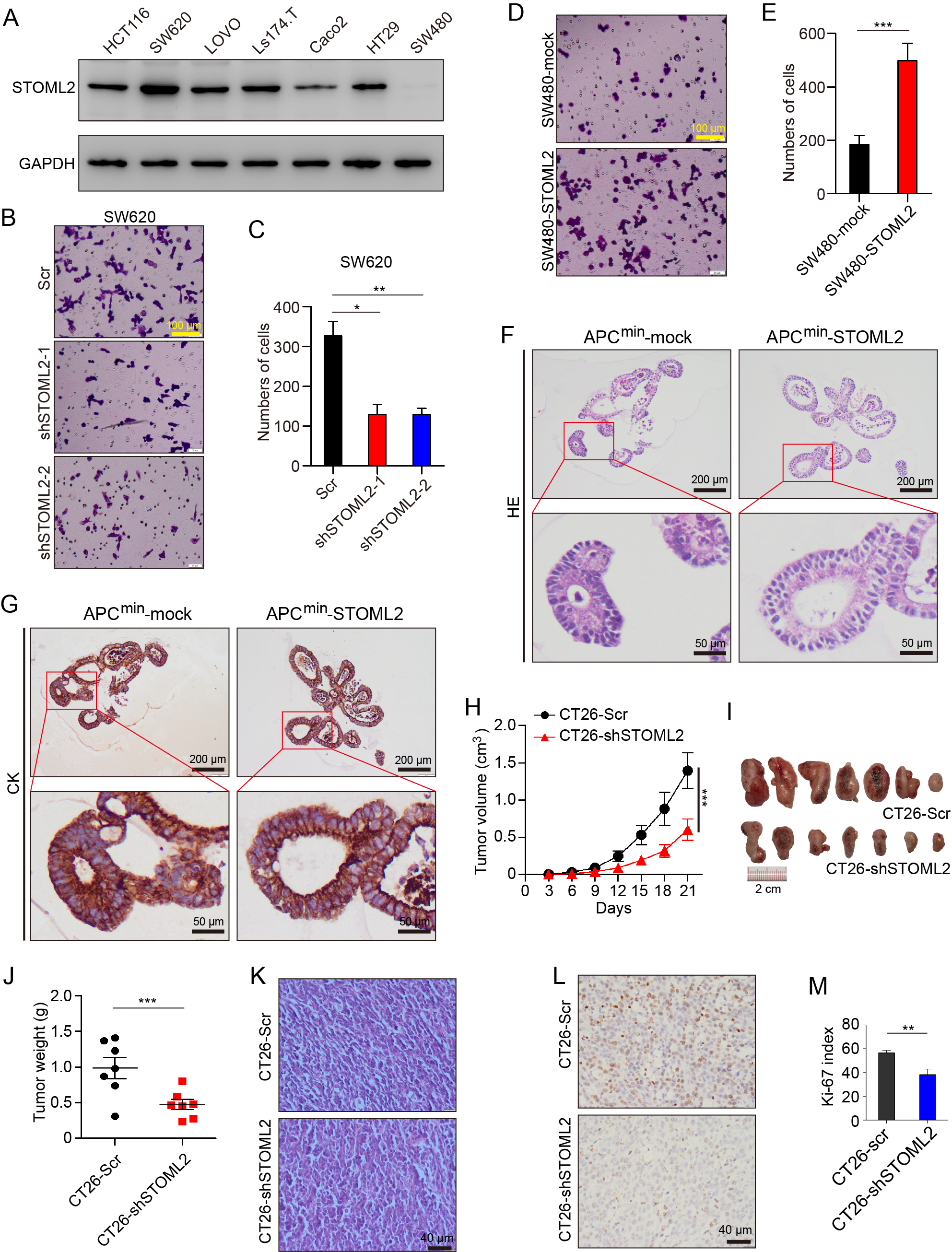

Supplement: Supplementary file 2 — Additional file 2. [file 13046_2021_2116_MOESM2_ESM.tif]

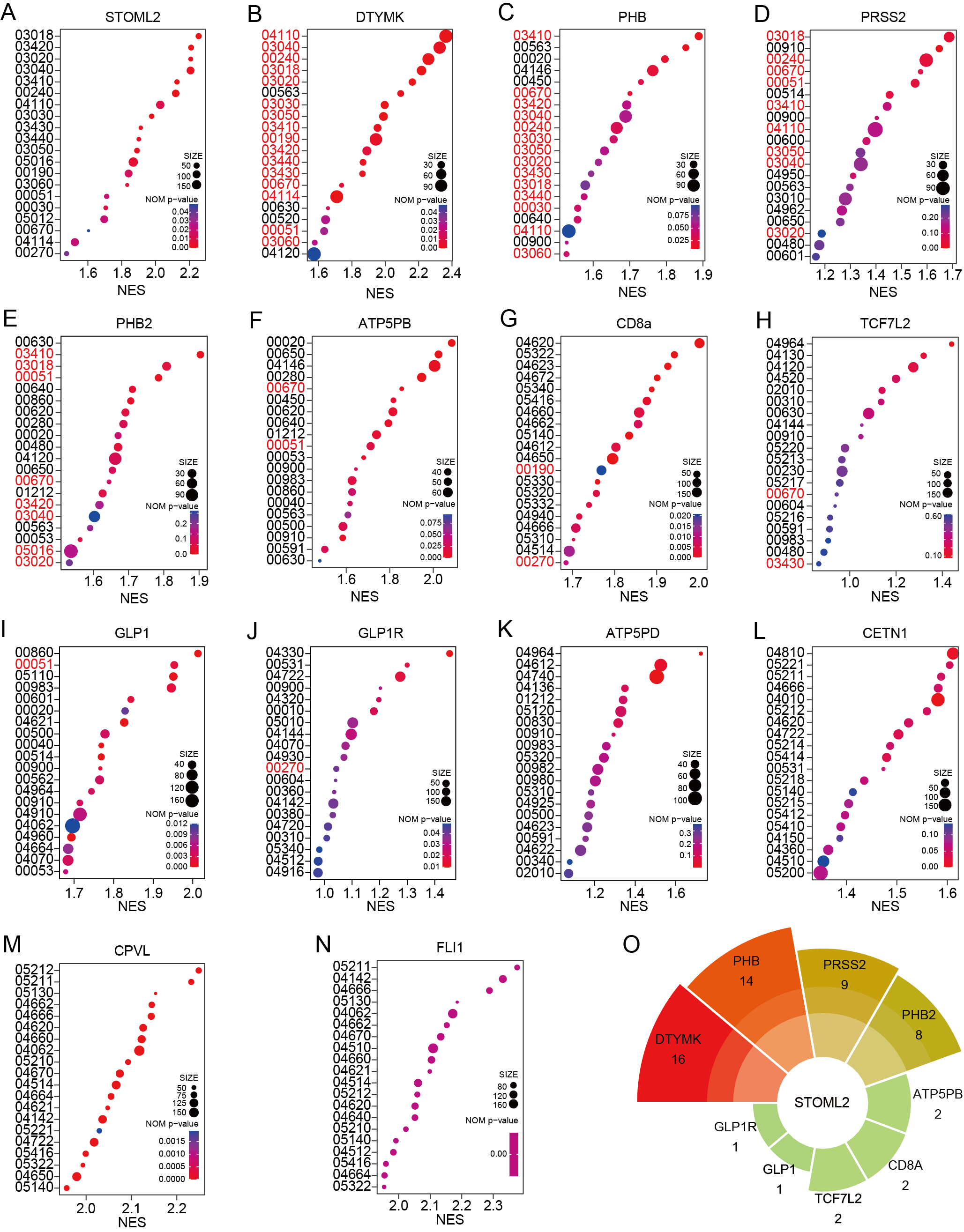

Supplement: Supplementary file 3 — Additional file 3. [file 13046_2021_2116_MOESM3_ESM.tif]

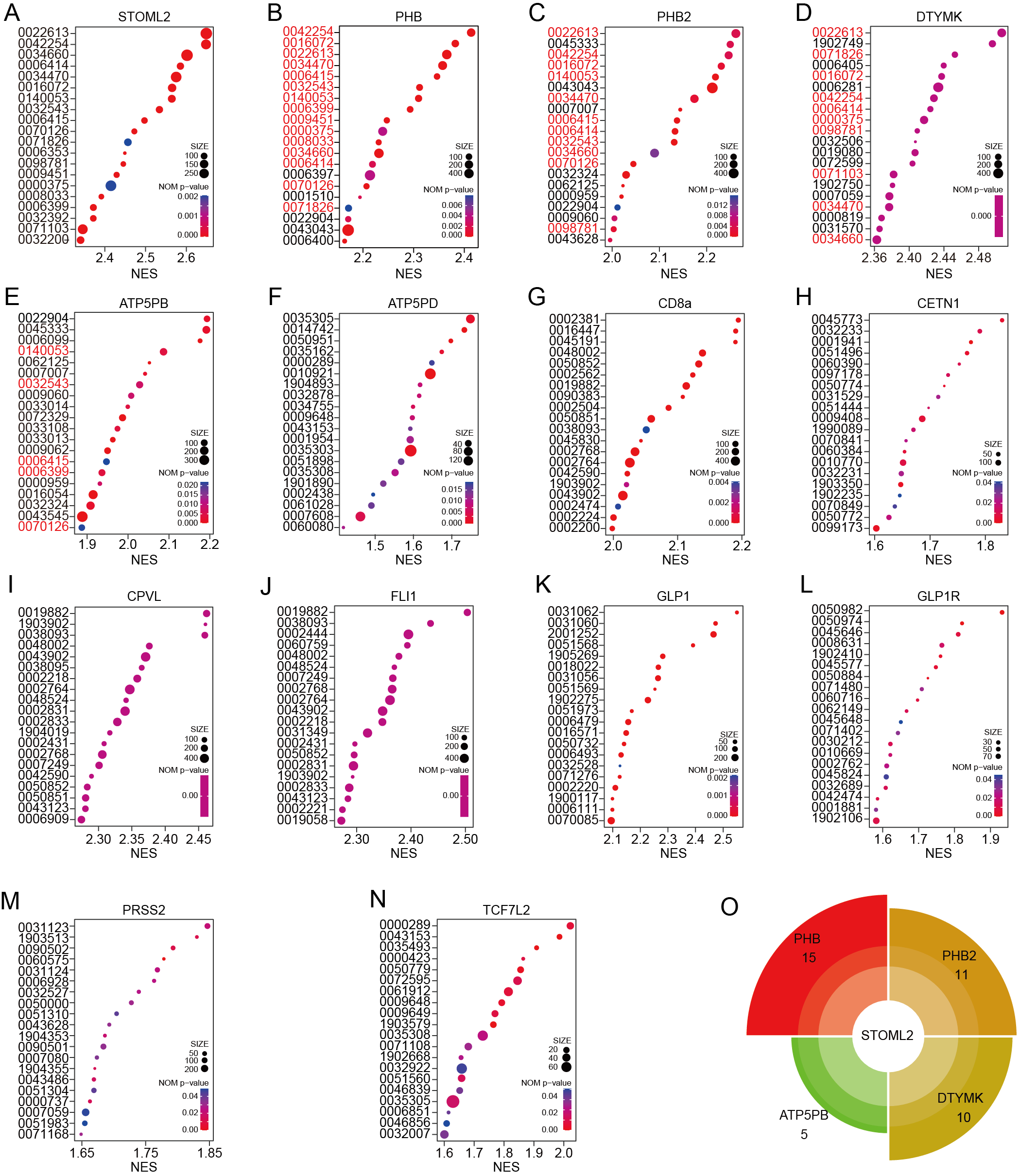

Supplement: Supplementary file 4 — Additional file 4. [file 13046_2021_2116_MOESM4_ESM.tif]

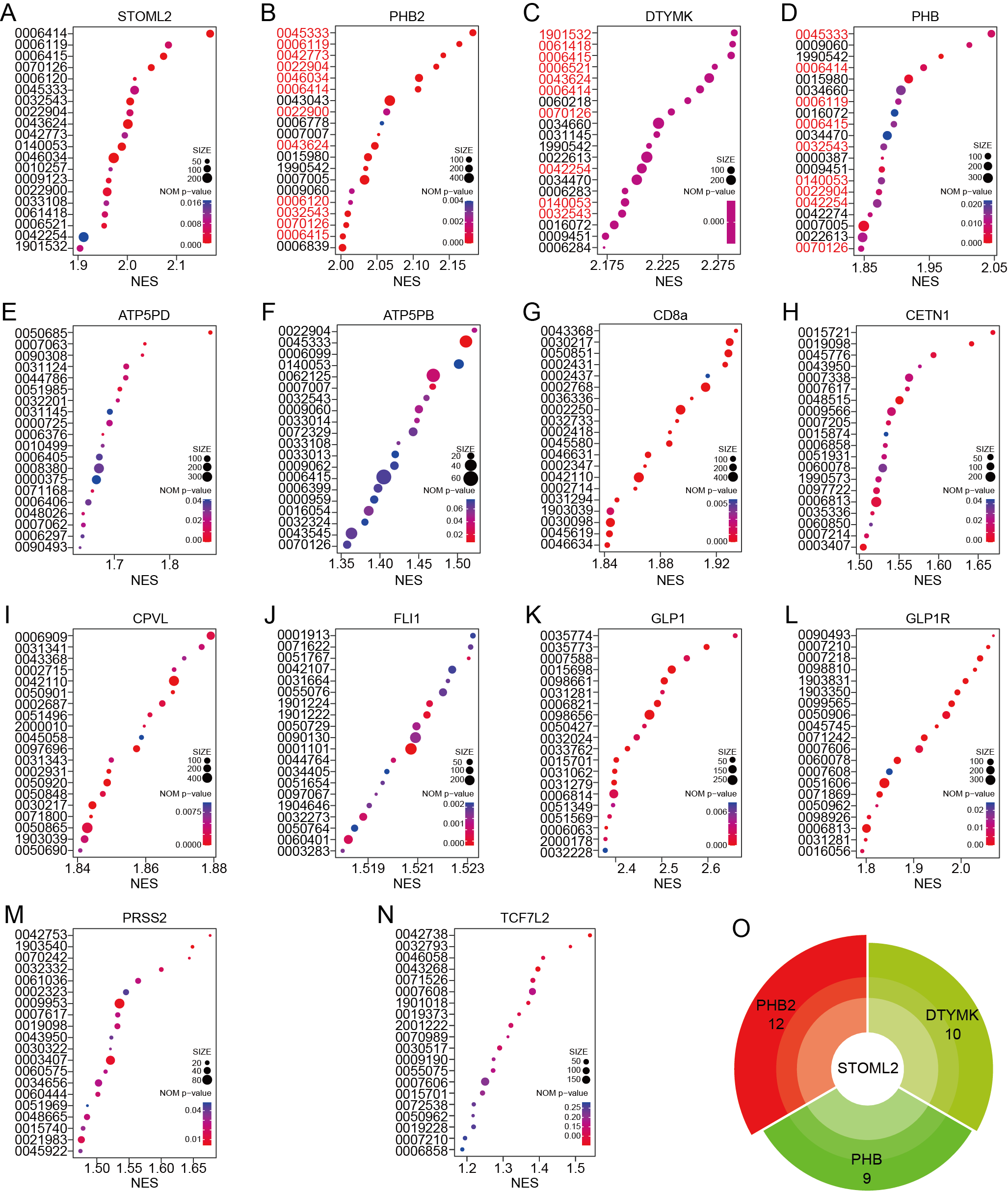

Supplement: Supplementary file 5 — Additional file 5. [file 13046_2021_2116_MOESM5_ESM.tif]

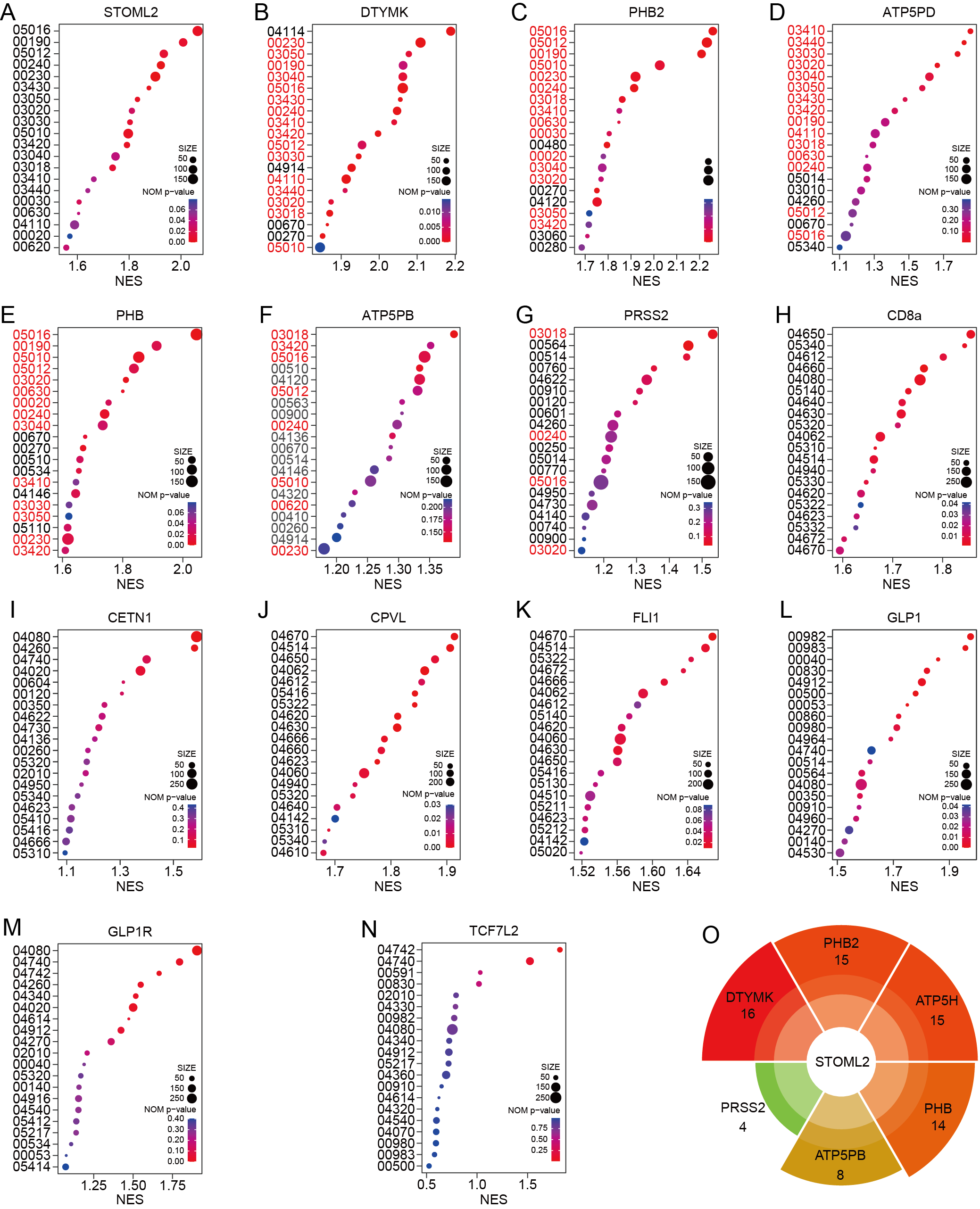

Supplement: Supplementary file 6 — Additional file 6. [file 13046_2021_2116_MOESM6_ESM.tif]

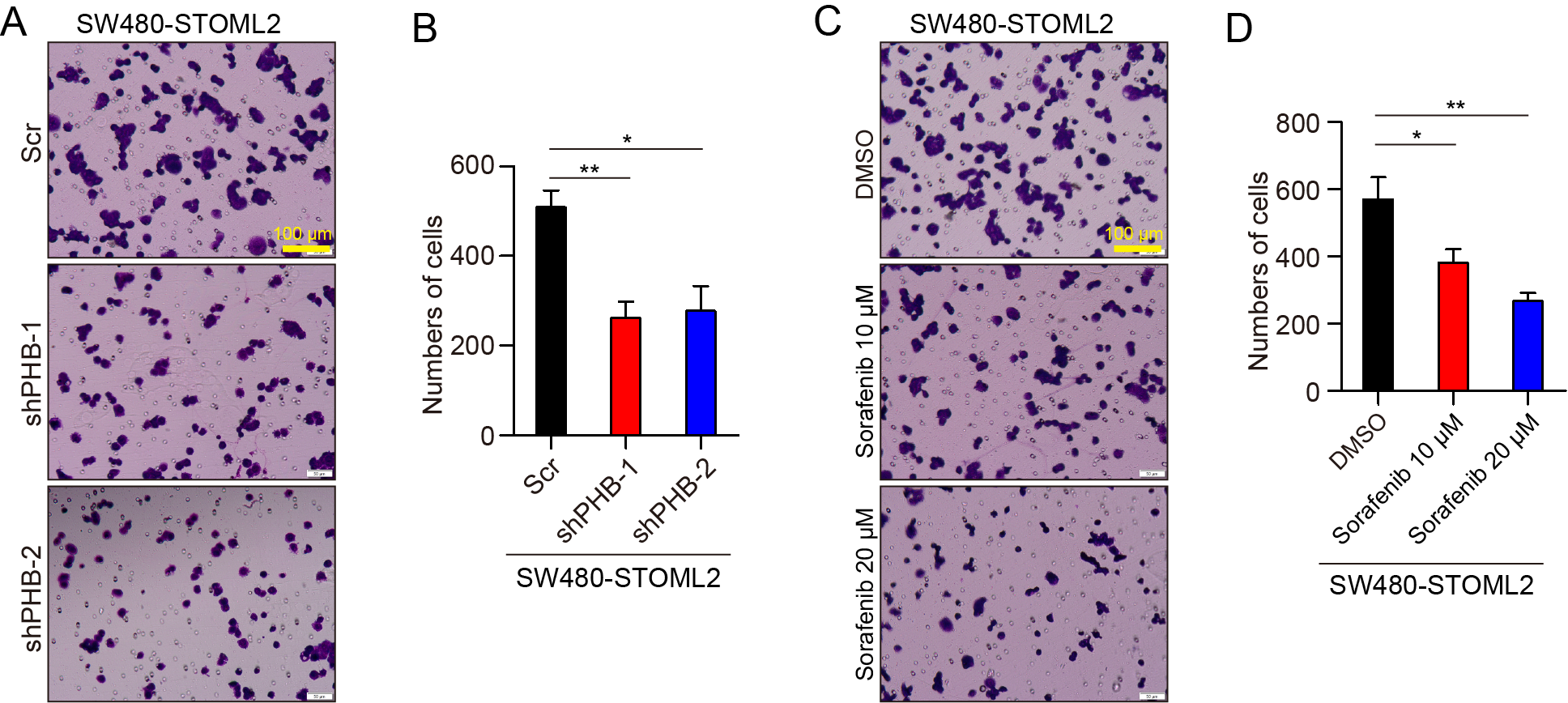

Supplement: Supplementary file 7 — Additional file 7. [file 13046_2021_2116_MOESM7_ESM.tif]
